# Longitudinal changes in oral conditions and oral candidiasis in palliative care inpatients: a longitudinal observational study

**DOI:** 10.3389/fdmed.2026.1831411

**Published:** 2026-07-02

**Authors:** Taeko Fukutani, Arisu Shibata, Yuki Okazaki, Chie Okugawa, Shunki Izumi, Seiya Hayashi, Yoji Nakase, Yasutaka Ishida, Tomoaki Hamana, Souichi Yanamoto, Kosei Okamoto

**Affiliations:** 1 Department of Dentistry and Oral Surgery, Hiroshima City North Medical Center Asa Citizens Hospital, Hiroshima, Japan; 2 Department of Oral Oncology, Graduate School of Biomedical and Health Sciences, Hiroshima University, Hiroshima, Japan; 3 Department of Dentistry and Oral Surgery, JA Onomichi General Hospital, Onomichi, Japan; 4 Department of Oral Surgery, Hiroshima Red Cross & Atomic-Bomb Survivors, Hiroshima, Japan

**Keywords:** longitudinal studies, oral candidiasis, oral health, oral health assessment tool (OHAT), oral hygiene, palliative care, prognosis, terminal care

## Abstract

**Background:**

Oral health influences comfort, communication, and quality of life in palliative care. However, its clinical drivers and prognostic significance remain poorly understood. This study examined longitudinal changes in oral conditions over time and identified factors associated with oral deterioration or improvement.

**Methods:**

A longitudinal observational study was conducted involving 300 inpatients receiving palliative care. Each patient underwent routine oral assessments using the Oral Health Assessment Tool (OHAT) both before and after oral care. Clinical characteristics, functional status activities of daily living (ADL), number of remaining teeth, oral care frequency, and outcomes at discharge were analyzed.

**Results:**

At baseline, 11.3% of the participants had oral candidiasis, which was associated with poorer oral health assessment scores. Among patients who subsequently died, the prevalence of candidiasis declined significantly after care, while oral health assessment scores worsened, particularly in the “tongue” and “saliva” categories. This paradoxical pattern may suggest a rapid shift in the microbiota immediately preceding death, despite oral care. In contrast, among patients who were discharged alive, candidiasis improved in 81.3% of affected individuals, and oral health assessment scores improved significantly. Post-care oral health assessment scores were influenced by activities of daily living, sex, and the number of remaining teeth. Patients with poorer baseline oral health assessment scores tended to show the greatest improvement, while those with initially better scores often deteriorated, possibly due to reduced care frequency.

**Conclusions:**

In palliative care, oral health is affected by activities of daily living, remaining teeth, sex, and the presence of candidiasis. Rapid intraoral decline near the end of life, coupled with reduced candidiasis, may reflect changes in oral conditions near the end of life; however, because no microbiological analyses were performed, this interpretation remains speculative. Longitudinal evaluation of functional status and oral conditions, potentially enhanced by artificial intelligence-based image analysis, may help clarify prognostic associations rather than provide definitive prognostic accuracy, and could support more tailored oral care.

## 1 Introduction

The World Health Organization emphasizes that palliative care should be accessible at all levels of healthcare (1). Patients receiving palliative care frequently experience oral complications such as severe oral mucositis (OM), xerostomia, and oral candidiasis. These conditions often result from deteriorating oral health and the side effects of multiple medications, including chemotherapy (2, 3).

However, oral assessment and treatment in this population remain largely unstandardized, making meaningful comparisons difficult (4). This inconsistency may stem partly from limited coordination between palliative medicine and dental care services (5, 6). Only a few studies have reported dental interventions in palliative care settings (7). For example, Watt et al. observed that dentistry often remains separate from mainstream healthcare (8). Similarly, Venkatasalu et al. highlighted the neglect of oral health in palliative patients (9), while Lobbezoo et al. advocated for its integration into palliative care practice (10). Notably, over 40% of patients receiving palliative care are unable to communicate their oral hygiene needs (3).

Patients in the terminal phase of illness present with diverse conditions and clinical backgrounds, which complicates dental management. During active treatment, the goal of palliative care is to ensure the safe completion of disease-directed therapies. In this context, dentistry focuses on managing OM, taste disorders from chemotherapy and radiotherapy, and oral candidiasis. For patients at the end of life, the primary purpose of oral care shifts towards alleviating pain and preserving oral function, ultimately enhancing comfort and quality of life. Supporting patients in maintaining speech and communication with family and medical staff remains essential to this objective (11).

Despite its importance, there is a lack of longitudinal studies tracking oral changes near the end of life. Most existing studies are cross-sectional, and only a limited number have evaluated temporal changes in oral health during the terminal phase (12). Moreover, how oral conditions evolve in the final days remains largely unknown (13).

Since the Palliative Care Center was established at JA Onomichi General Hospital in March 2020, collaboration between the Department of Palliative Care and the Department of Dentistry and Oral Surgery has been strengthened to deliver more integrated oral care. In this study, we examined longitudinal changes in oral conditions among palliative care patients. We aimed to clarify differences between patients who died and those discharged alive, and to identify factors associated with oral deterioration in patients who died and oral improvement in those who survived.

## 2 Materials and methods

### 2.1 Patient referral

At JA Onomichi General Hospital, physicians and nurses in the Palliative Care Center perform an initial oral assessment, including oral cleanliness, oral candidiasis, and oral mucositis (OM). Patients requiring professional oral management are referred to the Department of Dentistry and Oral Surgery, where dentists and dental hygienists perform detailed oral examinations. These professionals further evaluate oral cleanliness, oral candidiasis, and OM. Oral candidiasis is first diagnosed based on clinical findings, such as removable white plaques, erythematous mucosa with erosions, and pseudomembranous lesions. When the clinical diagnosis is uncertain, a sterile swab is used to collect samples from the affected mucosa, which are submitted for microbiological testing. The presence of Candida species on culture supports the diagnosis. Samples were processed by the hospital laboratory using standard fungal culture procedures on Sabouraud agar, and culture results were used to support the diagnosis when clinical findings were inconclusive. Based on these assessments, an individualized oral care plan is developed for each patient.

### 2.2 Assessors and reliability

All assessors were trained in the use of the Oral Health Assessment Tool. Training included instruction using standardized reference photographs and supervised practice sessions to ensure consistent scoring. To ensure scoring consistency, dentists and dental hygienists regularly calibrated their assessments through periodic consensus meetings in which sample cases were jointly reviewed. For each patient, the same assessor performed both the pre-care and post-care evaluations to minimize inter-rater variability.

### 2.3 Study design

This longitudinal study was conducted from January 2021 to December 2023 and included 300 inpatients with malignant tumors who were referred to the Department of Dentistry and Oral Surgery at JA Onomichi General Hospital/Palliative Care Center. During the first dental visit, dentists and dental hygienists assessed the oral cavity using the Oral Health Assessment Tool (OHAT). OHAT is a validated oral screening form developed by Chalmers et al. (14). It evaluates eight categories of oral problems: lips, tongue, gums, saliva, natural teeth, dentures, oral cleanliness, and dental pain. Each category is assessed on a three-point scale: healthy (0 points), changes (1 point), and unhealthy (2 points). OHAT is widely recognized for its high validity and reliability (15).

#### 2.3.1 Participant grouping

**Figure 1:**
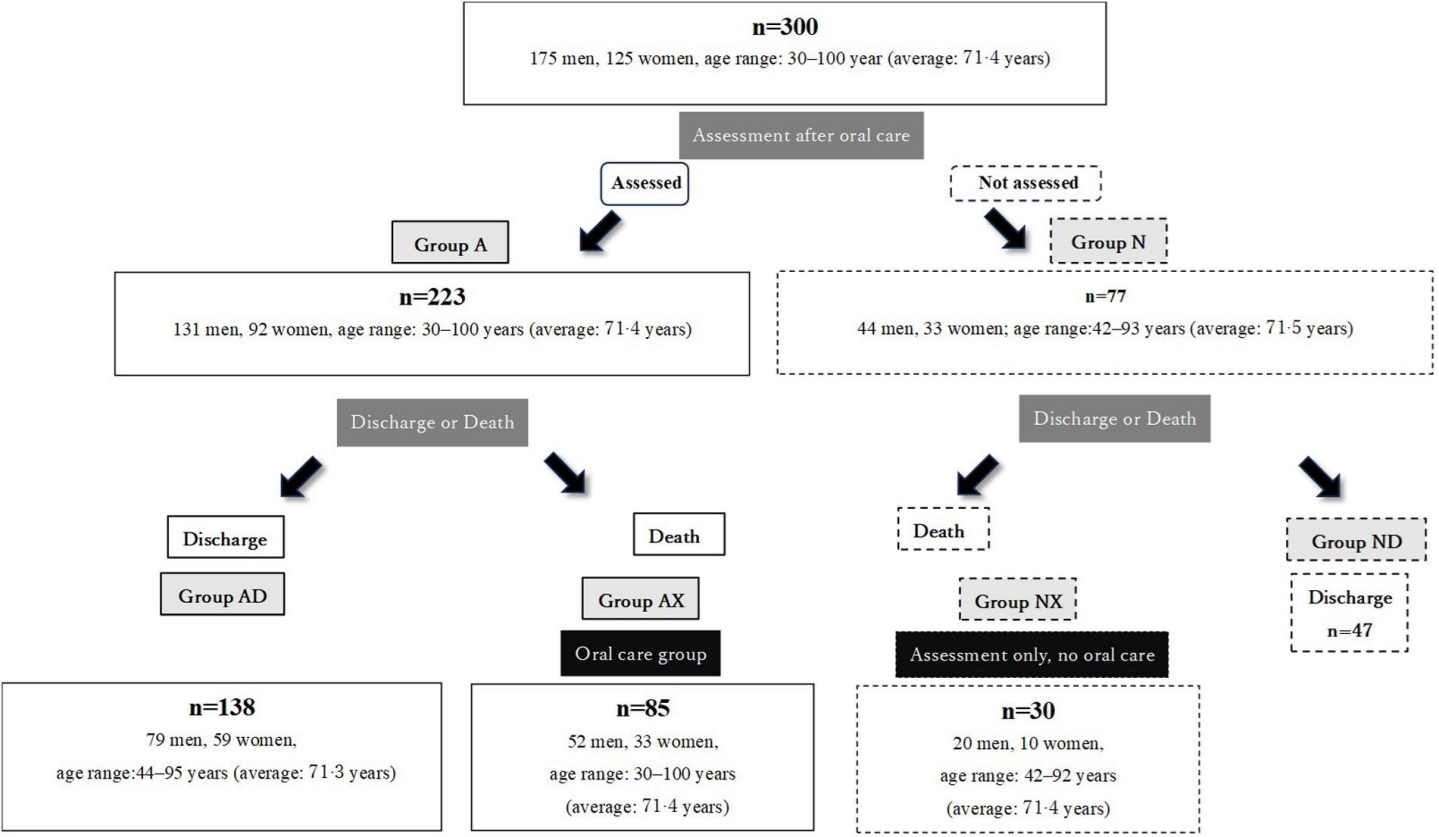
Study design.

As shown in Figure 1 and detailed in Supplementary Data 1 through 4, all 300 participants received an initial oral assessment. Based on whether oral care and follow-up evaluations could be completed, participants were categorized into two primary groups.

Participants who received oral care and completed both pre- and post-care assessments were categorized as Group A (assessed). Participants in Group A who were discharged alive were labeled Group AD, while those who subsequently died were labeled Group AX.

Participants who received an initial oral assessment but could not receive post-care evaluation due to sudden discharge, transfer, or rapid clinical deterioration were categorized as Group N (not assessed for follow-up). Among the participants in Group N, those who were discharged alive were labeled as Group ND, and those who died were labeled Group NX.

This hierarchical coding system (A → AD/AX; N → ND/NX) is used consistently throughout the manuscript.

#### 2.3.2 Statistical analysis

Statistical analyses were conducted according to the type and distribution of each variable. For non-normally distributed variables, the Mann–Whitney *U* test was used for comparisons between independent groups, and the Wilcoxon signed-rank test was applied for paired comparisons before and after oral care. Fisher's exact test was used to evaluate changes in oral candidiasis status. Multiple regression analysis was conducted to explore factors associated with oral health outcomes. These include oral candidiasis age, sex, oral care schedule, activities of daily living (ADL), number of remaining teeth, nutritional intake route, and clinical outcome. For these analyses, categorical covariates were coded as binary variables: the presence of oral candidiasis (presence = 1, absence = 0), sex (men = 1, women = 0); ADL status (requiring assistance = 1, independent = 0); number of remaining teeth (≤19 teeth = 1, ≥20 teeth = 0); nutritional intake route (non-oral intake = 1, oral intake = 0) and clinical outcome (death = 1, discharge = 0). A cut-off of 20 remaining teeth was applied based on previous surveys of masticatory ability and nutrition (16). A two-sided *p*-value of less than 0.05 was considered statistically significant. All analyses were performed using Statcel (4th edition). Prior to the multiple regression analysis, the assumptions of multicollinearity, normality, and homoscedasticity of residuals were checked and verified to be within acceptable limits. There were no missing data for any of the variables analyzed across the 300 included participants.

#### 2.3.3 Assessment timing

OHAT was performed at the initial dental visit before oral care (pre-care). The post-care assessment was conducted at the completion of the bedside oral care plan. The completion time varied depending on the patient's clinical condition, including situations in which transfer or discharge had been decided. For patients in Group AX, the post-care assessment was conducted when the palliative care team clinically diagnosed the patient as having entered the active terminal phase (end-of-life status), characterized by objective signs of imminent death such as a rapid decline in consciousness (Palliative Prognostic Index score deterioration) or a profound decrease in physical tolerance, rather than at a fixed chronological interval.

### 2.4 Oral care planning

Oral care frequency was not standardized because care plans were individualized according to each patient's clinical condition, tolerance, and goals of care. In palliative care settings, patients often experience rapid fluctuations in consciousness, discomfort, or physical burden, making fixed-interval oral care protocols impractical. As a result, care was provided daily, every 2–3 days, or less frequently depending on patient needs and feasibility.

Dental hygienists provide approximately 20 min of bedside oral care per visit, in accordance with individualized care plans. This care continues consistently through the patient's stay, including post-care reassessment. Toothbrushes are used for oral hygiene, and sponge brushes are used for tongue care. Xerostomia is managed with Weltec's ConCool® mouth gel and rinse, or Refrecare Spray. Vaseline is applied to the lips four times daily to address dryness.

For oral candidiasis, miconazole gel (200–400 mg/day) is administered after meals and at bedtime for seven days. If miconazole is contraindicated, amphotericin B syrup (50–100 mg/day) is given for six days. Although amphotericin B is typically used parenterally, the syrup formulation is widely used in Japan as a topical oral antifungal agent. Treatment for OM depends on symptoms and may include steroid ointment, wound-covering hydrogels such as Episil® oral liquid, or sodium sulfonate hydrate mouthwash containing xylocaine.

### 2.5 Ethics approval

This study was consistent with the Declaration of Helsinki. Approval was granted by the Hiroshima City North Medical Center Asa Citizens Hospital Ethics Committee (approval no. 06-5-26). Verbal informed consent was obtained from all participants. Furthermore, the authors did not use generative AI in the writing of this manuscript, including both textual and visual content.

## 3 Results

### 3.1 Oral condition at first visit

Participants' characteristics are detailed in Supplementary Data 1. At the initial visit to the department, 34 of 300 participants (11.3%) presented with oral candidiasis. Of these, only five (14.7%) were referred for suspected oral candidiasis, suggesting that approximately 85% of cases were not identified at the time of referral.

Oral candidiasis correlated with OHAT scores. The Mann–Whitney *U* test revealed a significant difference in total OHAT scores between participants with or without candidiasis (*z* = 1.64, *p* = 0.036). Participants with poorer oral health had a higher incidence of candidiasis.

### 3.2 Oral health before and after oral care

Participants' characteristics appear in Supplementary Data 2. Multiple regression analysis results are shown in Table 1. Before oral care, age was independently associated with candidiasis. Over 80% of participants were aged 70 years or older, and the incidence of candidiasis increased with age (Table 2).

**Table 1:** Multiple regression analysis of factors associated with the presence of oral candidiasis based on pre-oral care assessments.

Predictor variable	Coefficient	Standard error	*t*	*p*	95% Confidence interval
Age	0.0052	0.0021	2.49	**0**.**014**	0.0011 to 0.0094
Sex	0.044	0.046	0.96	0.34	−0.046 to 0.13
Outcome	0.036	0.047	0.77	0.44	−0.056 to 0.13
Schedule (Day)	0.0012	0.0027	0.43	0.67	−0.0042 to 0.0066
Schedule (Times)	−0.00085	0.014	−0.060	0.95	−0.029 to 0.027
Schedule (Frequency)	0.00038	0.0013	0.2991	0.77	−0.0021 to 0.0029

Bold values indicate statistically significant differences (*p* < 0.05).

**Table 2:** Multiple regression analysis of predictors of oral candidiasis after oral care.

Predictor variable	Coefficient	Standard error	*t*	*p*	95% Confidence interval
Age	0.0031	0.0016	1.92	0.056	−0.000075 to 0.0063
Sex	0.039	0.035	1.11	0.27	−0.030 to 0.11
Outcome	−0.038	0.036	−1.06	0.29	−0.109 to 0.033
Schedule (Day)	−0.00024	0.0021	−0.12	0.91	−0.0044 to −0.0039
Schedule (Times)	−0.0088	0.011	−0.81	0.42	−0.030 to 0.013
Schedule (Frequency)	−0.0011	0.0010	−1.10	0.27	−0.0030 to 0.00085

Mean OHAT scores before and after oral care were comparable, indicating no overall improvement in oral health following care (Supplementary Data 5).

Multiple regression analysis showed a significant association between clinical outcomes and OHAT scores (*p* = 0.00023; Table 3). We then compared the outcomes of the group whose OHAT scores improved and those whose scores worsened; of the improved group, 30.4% died, while 61.3% of the worsening group died. The average OHAT scores for discharged and deceased participants were 1.98 and 3.18, respectively, indicating significantly better oral health among discharged participants (p < 0.0001).

**Table 3:** Multiple regression analysis of factors associated with improvement in OHAT scores.

Predictor variable	Coefficient	Standard error	*t*	*p*	95% Confidence interval
Oral candidiasis	0.35	0.29	1.19	0.24	−0.23 to 0.93
Outcome	−0.74	0.26	−2.80	**0**.**0055**	−1.26 to −0.22
Sex	−0.07	0.26	−0.28	0.78	−0.58 to 0.44
Age	−0.01	0.012	−0.55	0.58	−0.030 to 0.017
Schedule (Day)	0.01	0.015	0.57	0.57	−0.022 to 0.039
Schedule (Times)	−0.12	0.080	−1.54	0.13	−0.28 to 0.035
Schedule (Frequency)	0.00	0.0072	0.13	0.90	−0.013 to 0.015

Bold values indicate statistically significant differences (*p* < 0.05).

### 3.3 Oral health of participants who died (group AX)

Participant characteristics are listed in Supplementary Data 3. In Group AX, the prevalence of oral candidiasis declined significantly from 15.3% before oral care to 4.7% after care (relative reduction: 69.3%), as confirmed by Fisher's exact test (*p* = 0.019).

Contrary to expectations, total OHAT scores increased from 2.75 before oral care to 3.18 after care, indicating worsening oral health. This suggests that in participants nearing death, oral conditions were less likely to improve even with intervention. The Wilcoxon signed-rank test showed significant deterioration in the “tongue” score (*z* = −3.15, *p* = 0.0016) and “saliva” score (*z* = −4.11, *p* < 0.0001).

### 3.4 Comparison of end-of-life oral health between care (group AX) and non-care groups (group NX)

The prevalence of oral candidiasis in Group NX (no oral care) was 6.7%, compared to 4.7% in Group AX (oral care), with no significant difference (Supplementary Data 6). This finding was unexpected. Although Group AX showed a 69.3% relative reduction in candidiasis from before to after oral care, this improvement was not solely attributable to oral care. The results suggest that candidiasis may decrease shortly before death even among patients receiving oral care.

Total OHAT scores were similar between Groups AD and AX (3.18 vs. 3.17), indicating no significant oral health benefit of care just before death. However, Mann–Whitney *U* tests showed Group AX had significantly worse “natural teeth” scores than Group NX (0.26 vs. 0.067; *z* = 1.64, *p* = 0.034), indicating improved condition of remaining teeth in participants who received care.

### 3.5 Oral health of discharged participants (group AD)

Comparisons of pre- and post-oral care assessments in Group AD are detailed in Supplementary Data 4.

#### 3.5.1 Oral candidiasis

Before oral care, 16 participants (11.6%) had oral candidiasis. After care, oral candidiasis was completely resolved in 13 of these cases (81.3%). However, nine additional cases (6.5%) were newly diagnosed during the oral care period despite having no signs at baseline.

#### 3.5.2 Oral health assessment tool scores

The Wilcoxon signed-rank test showed a significant difference in OHAT scores before and after oral care (*Z* = −1.898, *p* = 0.029). Multiple regression analysis indicated that pre-care OHAT scores were influenced by oral care schedule, ADL, and number of remaining teeth (Table 4). Post-care OHAT scores were associated with sex and number of remaining teeth (Table 5).

**Table 4:** Multiple regression analysis of pre-care OHAT (group AD).

Predictor variable	Coefficient	Standard error	*t*	*p*	95% Confidence interval
Age	−0.031	0.019	−1.626	0.106	−0.069 to 0.007
Sex	−0.679	0.380	−1.786	0.076	−1.431 to 0.073
Schedule (Day)	−0.032	0.021	−1.547	0.124	−0.073 to 0.009
Schedule (Times)	0.260	0.120	2.175	**0**.**031**	0.024 to 0.497
Schedule (Frequency)	0.002	0.011	0.167	0.868	−0.020 to 0.023
ADL	1.135	0.385	2.950	**0**.**004**	0.374 to −1.897
Number of remaining teeth	−0.055	0.022	−2.522	**0**.**013**	−0.098 to −0.012
Nutrition route (oral intake)	0.727	0.762	0.954	0.342	−0.781 to 2.234

Bold values indicate statistically significant differences (*p* < 0.05).

**Table 5:** Multiple regression analysis of post-care OHAT scores (group AD).

Predictor variable	Coefficient	Standard error	*t*	*p*	95% Confidence interval
Age	−0.027	0.018	−1.508	0.134	−0.062 to 0.0080
Sex	−0.774	0.352	−2.198	**0**.**030**	−1.471 to −0.077
Schedule (Day)	−0.025	0.019	−1.281	0.203	−0.063 to 0.013
Schedule (Times)	0.170	0.111	1.529	0.129	−0.050–0.39
Schedule (Frequency)	0.013	0.010	1.301	0.195	−0.007 to 0.033
ADL	0.519	0.357	1.456	0.148	−0.186–1.23
Number of remaining teeth	−0.047	0.020	−2.359	**0**.**020**	−0.087 to −0.0080
Nutrition route (oral intake)	0.616	0.706	0.872	0.385	−0.781 to 2.01

Bold values indicate statistically significant differences (*p* < 0.05).

#### 3.5.3 Sex differences

Sex differences were evaluated using the Mann–Whitney *U* test. Before oral care, women had significantly drier lips than men (mean score: 0.32 vs. 0.13; *z* = 1.96, *p* = 0.013). However, after oral care, no statistically significant difference in lip dryness was observed between women and men (mean score: 0.18 vs. 0.09; *z* = 1.96, *p* = 0.066), indicating greater improvement among women.

Conversely, men had significantly poorer oral cleanliness than women both before (mean score: 0.77 vs. 0.45; *z* = 1.96, *p* = 0.0055) and after oral care (mean score: 0.60 vs. 0.37; *z* = 1.96, *p* = 0.066), indicating that men were less likely to improve. Men also showed smaller improvements in total OHAT scores (0.26 vs. 0.38).

#### 3.5.4 Oral care schedule

Participants with below-average pre-care OHAT scores had an average of 3.8 oral care visits, whereas those with above-average scores had 4.2 visits, indicating more frequent care for those with better oral health.

**Figure 2:**
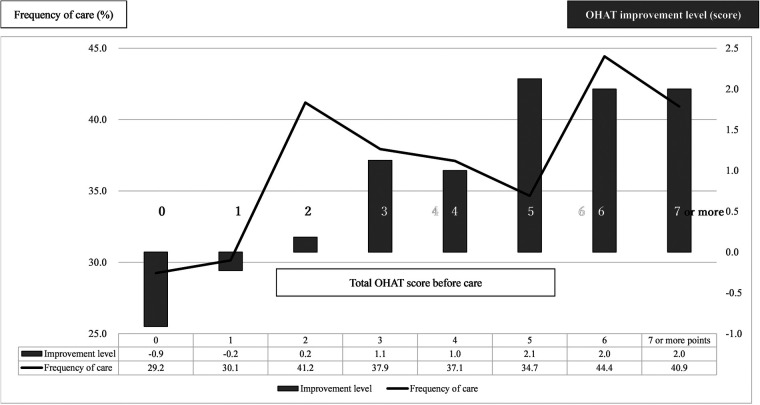
Assessment of oral care schedule. The worse the OHAT assessment before care, the greater the improvement and the more frequent the care.

Participants with initial OHAT scores of 0 or 1 often worsened after care. Those scoring 2 improved by 0.18 points, while those scoring 5 improved by 2.13 points. Greater initial impairment predicted greater improvement. Oral care frequency corresponded to initial OHAT severity (Figure 2).

#### 3.5.5 Activities of daily living

Pre-care OHAT scores were significantly associated with ADL status. Using the Mann–Whitney *U* test, significant differences were observed in the total OHAT score (*Z* = 3.21, *p* = 0.0013), “tongue” (*Z* = 2.35, *p* = 0.019), “gums” (*Z* = 2.37, *p* = 0.019), and “saliva” (*Z* = 2.92, *p* = 0.0035).

Participants with independent ADL had lower mean OHAT scores (1.69) than those requiring assistance (2.81). Subscores also indicated better oral health among independent participants.

After oral care, no significant differences in OHAT scores were observed between ADL groups, suggesting that oral care benefitted participants requiring assistance. Mean improvement in OHAT score was greater in the assistance-required Group AD (0.57) than in the independent group (0.016), indicating greater improvement among those who required assistance.

#### 3.5.6 Number of remaining teeth

Fifty-eight participants (42.0%) had 19 or fewer teeth, whereas 80 participants (58.0%) had 20 or more. Pre-care mean OHAT scores for those with 19 or fewer teeth and those with 20 or more were 2.67 and 2.01, respectively, indicating that participants with a greater number of remaining teeth had better oral health. Post-care scores for the two groups were 2.40 and 1.68, respectively. Although the level of improvement was similar between groups (0.72 vs. 0.66), participants with fewer remaining teeth consistently exhibited poorer oral health, even after receiving oral care.

## 4 Discussion

The OHAT did not show significant changes before and after oral care across all patients, suggesting that its utility may be limited in palliative care settings, rather than indicating that oral care itself was ineffective. However, among patients who were discharged alive, oral status improved significantly after care, which indicates a beneficial effect. This discrepancy highlights several limitations of the OHAT when applied to end-of-life settings.

Because palliative care is recommended throughout the disease trajectory, from diagnosis and active treatment to the final days of life, the term “end of life” represents a heterogeneous clinical stage. Some patients remain relatively stable and close to the treatment phase, whereas others are in the final hours or days before death. This variability creates a dilemma for clinicians, as the goals, feasibility, and expected benefits of oral care differ substantially across these stages. This heterogeneity of end-of-life trajectories also emphasizes the importance of considering prognosis when evaluating oral conditions in patients receiving palliative care.

### 4.1 Prognostic implications and outcomes at discharge

Patients who died had significantly worse oral health at the time of death than those discharged alive. These findings support integrating prognostic considerations into oral health assessments for patients receiving palliative care. For patients who were very close to death, oral care appeared to have limited observable effects on tongue and saliva parameters. Deterioration in these domains often outpaced the benefits of care, likely due to reduced self-cleansing ability.

Previous studies have reported that oral care can improve oral cleanliness, moisture, and comfort even in patients approaching the end of life (17–21). Additionally, studies using OHAT and the Oral Assessment Guide have reported improvements in oral health among terminally ill patients (22, 23). At first glance, these findings may appear inconsistent with our observation that oral status continued to deteriorate in patients who died. However, several methodological and clinical differences may explain this discrepancy. Many prior studies focused on short-term improvements in specific aspects of oral hygiene or subjective comfort, whereas our study assessed broader oral health parameters using the OHAT and followed patients through to the point of death. Moreover, our cohort included a substantial proportion of patients in the final days of life, when systemic decline and reduced self-cleansing capacity may outweigh the benefits of routine oral care. Thus, our findings do not contradict the existing literature but rather suggest that the measurable impact of oral care may diminish as death approaches.

Poor oral health is associated with halitosis, which can interfere with meaningful social interaction at the end of life. For this reason, oral care remains important. In this context, the aim should shift from improving oral status to maintaining a minimum standard that supports dignity and reduces caregiver burden. Previous research has shown that oral health declines rapidly in the final days of life, despite continued care (19, 20), and that changes in the oral cavity can predict death within seven days in patients with terminal cancer. Nakao et al. reported that OHAT had the strongest prognostic value at 21 days before death (24). If further validated, oral assessment may have prognostic relevance, although the present findings represent associations rather than predictive performance. Because no prognostic model was developed in this study, the potential prognostic utility of oral assessments should be considered exploratory. Given the OHAT's broad focus, future studies incorporating artificial intelligence–based analysis of intraoral images may help clarify prognostic associations and support more tailored oral care.

The interpretation of changes in oral health must also consider variability in the timing of assessments. Because pre- and post-care evaluations were conducted at different points in each patient's clinical trajectory, the interval between assessments was not standardized. This is particularly relevant for patients nearing the end of life, in whom oral conditions can deteriorate rapidly over a short period. As a result, some of the observed changes in OHAT scores or candidiasis status may reflect differences in assessment timing rather than the direct effects of oral care. Future studies with fixed or narrower assessment intervals are needed to clarify temporal patterns in oral deterioration.

### 4.2 Oral candidiasis

Timely identification and treatment of oral candidiasis are essential. In this study, 85% of cases were undiagnosed before dental evaluation. Due to its varied presentation, even dental professionals may miss diagnoses without specialized training (25).

Despite its opportunistic nature, candidiasis significantly improved with care. However, 6.5% of patients developed new infections, and older patients receiving palliative care remained especially vulnerable. The OHAT does not include a category for candidiasis, likely due to its diagnostic complexity. Nevertheless, including candidiasis as a consideration is crucial when planning treatment.

Among patients who subsequently died, oral candidiasis improved after receiving care. Although the prevalence appeared lower just before death, this finding should be interpreted with caution. Several alternative explanations may account for this pattern, including the effects of routine antifungal therapy, reduced clinical detectability due to xerostomia or decreased cooperation, and changes in oral intake or systemic status. Because microbiological analyses were not performed, we cannot draw conclusions regarding microbiota composition or biological mechanisms underlying this observation.

Interestingly, patients who died without receiving oral care (Group NX) had better scores for “natural teeth” than those who received care (Group AX). This category includes evaluations of dental caries and root remnants. This difference may reflect baseline oral conditions or care patterns rather than biological changes. However, when considering the biological plausibility of our findings, the simultaneous lower prevalence of both oral candidiasis and dental caries in the non-care group shortly before death implies a profound, systemic shift in the intraoral microenvironment and oral microbiota immediately preceding death. Near the end of life, drastic physiological changes, such as severe dehydration, complete cessation of oral intake, and extreme salivary depletion, fundamentally alter the intraoral substrate. Such a hostile environment may suppress not only Candida species but also cariogenic bacteria like Streptococcus mutans, which rely on dietary carbohydrates and moisture to thrive. Previous literature has reported complex interactions and symbiotic synergy between C. albicans and S. mutans within dental plaque biofilms (26, 27); therefore, a terminal collapse of this intraoral ecosystem might explain the synchronized decline in both infectious conditions. Nevertheless, our study design does not allow us to infer definitive microbiological shifts.

Participants in Group A were those who were clinically stable enough to receive oral care and complete both pre- and post-care assessments, whereas Group N included individuals who could not undergo follow-up due to sudden discharge, transfer, or rapid clinical deterioration. As a result, Group AX represents a subset of patients who remained sufficiently stable to receive repeated assessments, while Group NX includes patients with more rapid decline. This difference in clinical trajectories may have introduced selection bias, potentially influencing the observed reduction in candidiasis and the patterns of oral deterioration near the end of life.

In interpreting these findings, several potential confounding factors should be considered. Disease severity, comorbidities such as diabetes or immunosuppression, medication use including antibiotics, antifungals, and corticosteroids, and nutritional or hydration status may all influence oral conditions and the prevalence of candidiasis in palliative care patients. In particular, the routine use of antifungal therapy makes it difficult to distinguish whether changes in candidiasis reflect natural disease progression or treatment effects. Because these factors were not fully controlled for in our analyses, the observed associations should be interpreted with caution.

### 4.3 Sex differences in oral status

Overall, women demonstrated better oral health than men, although women had drier lips before care, possibly due to cosmetic use. Existing literature has identified sex- and gender-related disparities in oral health. For instance, Lipsky et al. (28) emphasized the need for research to understand these differences and improve prevention and treatment strategies, particularly for men. Sex should be considered in palliative oral health assessments.

### 4.4 Activities of daily living

Patients with ADL dependence showed greater improvement in oral health after care, indicating the need for proactive interventions in this group. Before receiving care, these individuals had worse assessments for “tongue,” “gums,” and “saliva” compared to those with independent ADL. Ninomiya & Hiratsuka (29) similarly reported poor oral health in patients with low ADL, including worse “lips,” “tongue,” and “saliva” assessments. Reduced mobility likely contributes to impaired oral muscle function and decreased salivary flow, leading to mucosal dryness. Although this study assessed ADL only at the initial visit, longitudinal evaluation of functional status may provide additional insights. Tracking changes in ADL over time could clarify how declining mobility influences oral health trajectories and the effectiveness of care interventions in patients receiving palliative care.

### 4.5 Number of remaining teeth

Patients with fewer remaining teeth had poorer baseline oral health and were less likely to show improvement after care. In a healthy oral environment, natural self-cleaning mechanisms depend on the interaction of teeth, saliva, food, and oral structures during mastication (30). Saliva also provides mucosal protection, facilitating tissue repair and reducing inflammation (31). Patients with more remaining teeth may retain these physiological benefits, whereas those with fewer teeth may require more frequent care.

### 4.6 Oral care scheduling

Patients with poor initial OHAT scores appeared to benefit most from care, while those with better baseline scores often experienced deterioration. Although this trend may reflect reduced care frequency due to assumptions about self-sufficiency, it may also be partly explained by regression to the mean, as individuals with more severe initial impairment tend to show greater numerical change on repeated assessments regardless of intervention. Basing care schedules solely on baseline status may therefore lead to both underestimation of deterioration and insufficient support for patients who appear stable at first assessment. Regular reassessment is essential to ensure that care intensity is adapted to changing conditions.

In this study, patients in the final days of life experienced limited observable benefit from oral care. This suggests that conventional goals and techniques may no longer be suitable during the terminal phase. For patients nearing death, it may be more appropriate to reframe oral care as part of end-of-life or post-mortem hygiene rather than routine oral management. Such a shift in focus could reduce the burden on healthcare providers while still preserving patient dignity.

### 4.7 Limitations

This study has several limitations. It was conducted at a single center, which may limit the generalizability of the findings. The observational design precludes causal inference regarding the relationship between oral care and oral health outcomes. The timing of pre- and post-care assessments varied depending on clinical conditions. Although assessors were trained and calibration meetings were held, residual assessor bias cannot be completely excluded. Missing data due to rapid clinical deterioration or early discharge may also have influenced the results.

In addition, although changes in oral candidiasis were observed, microbiological analyses were not performed for all participants. Therefore, we cannot draw conclusions regarding microbiota composition or the biological mechanisms underlying the decline in candidiasis near the end of life. Alternative explanations—such as the effects of antifungal therapy, reduced detectability due to xerostomia or decreased cooperation, and changes in oral intake or systemic status—should be considered when interpreting these findings.

Future multicenter studies with standardized assessment intervals, larger sample sizes, and microbiological evaluation are warranted to clarify the mechanisms underlying oral changes in palliative care patients.

Furthermore, several potential confounding factors—such as disease severity, comorbidities, medication use (including antibiotics, antifungals, and corticosteroids), and nutritional or hydration status—were not fully accounted for. These factors may have influenced both oral health outcomes and changes in candidiasis, limiting the interpretability of the findings.

Despite these limitations, this study may offer meaningful longitudinal observations of real-world oral changes in palliative care patients, potentially serving as a helpful clinical reference for end-of-life oral management.

### 4.8 Conclusions

Oral health in palliative care patients was associated with prognosis, oral candidiasis, sex, ADL, and the number of remaining teeth. These factors should be incorporated into oral assessments and treatment planning to more effectively tailor care to individual patient needs. Notably, rapid changes in oral flora and the intraoral environment observed near the end of life offer a minimally invasive indicator of imminent death. Future studies should incorporate longitudinal assessments of functional status, oral flora, and intraoral conditions to clarify the mechanisms underlying rapid oral deterioration near the end of life. The development of more sensitive assessment tools, including the possible integration of AI-based image analysis, may enhance prognostic accuracy and support individualized oral care strategies in palliative care settings.

## Data Availability

The original contributions presented in the study are included in the article/Supplementary Material, further inquiries can be directed to the corresponding author.
