# Supplementary material for: Longitudinal changes in oral conditions and oral candidiasis in palliative care inpatients: a longitudinal observational study
Source: Front Dent Med. 2026 Jul 2;7:1831411. doi: 10.3389/fdmed.2026.1831411 (PMC13372980; doi:10.3389/fdmed.2026.1831411)
Supplement: Supplementary file 1 [file Table1.docx]

Supplementary Material

# Supplementary Data:

## Table S1: Oral condition at first visit. Details of the 300 participants.

## Table S2: Oral health status before and after oral care. Details of the 223 participants (Group A).

## Table S3: Details of deceased participants’ oral health (Group ND, n= 85; Group NX, n=30).

## Table S4: Details of the oral health of the 138 discharged participants (Group AD).

## Figure S1: Comparison of oral health status before and after oral health care.

## Figure S2: Comparison of oral candidiasis prevalence before and after oral care in Group ND, and in Group NX (no care).

# Supplementary Figures


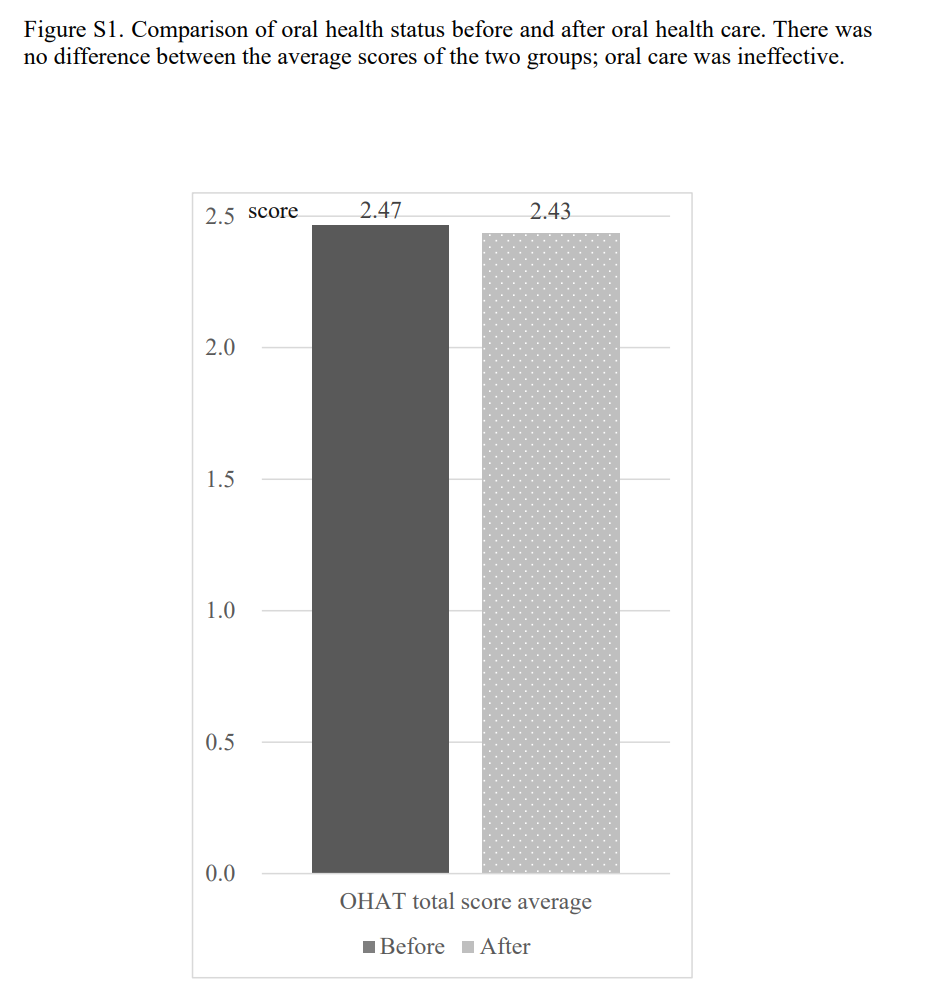


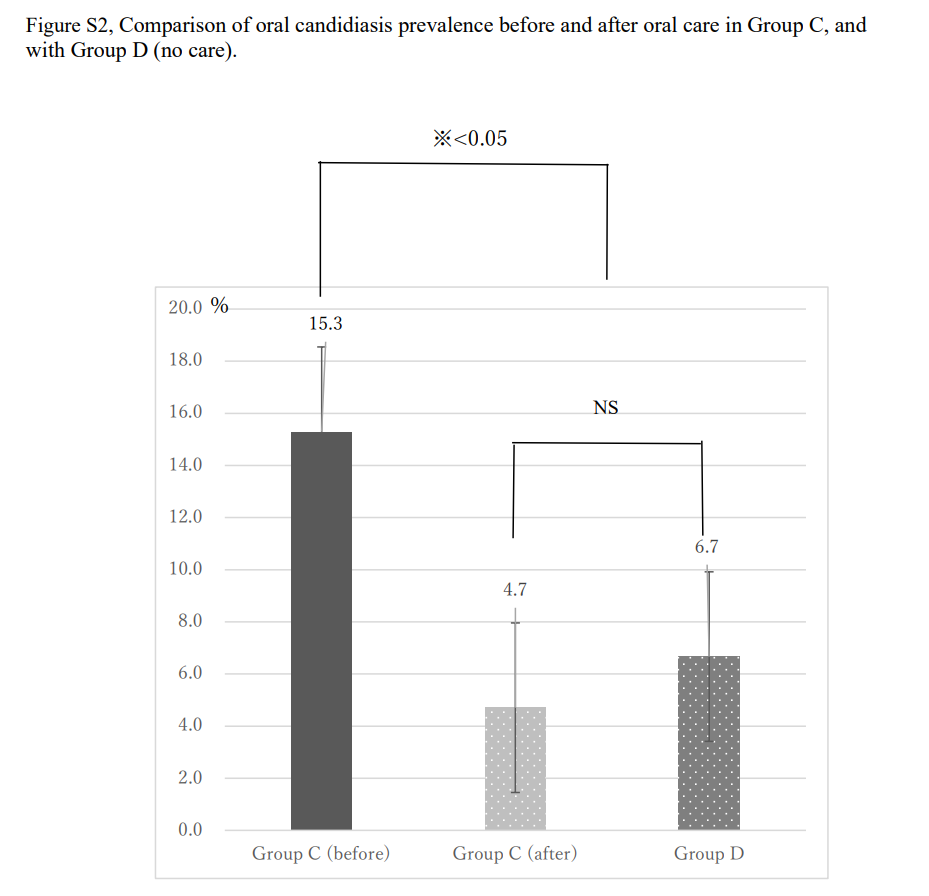


**
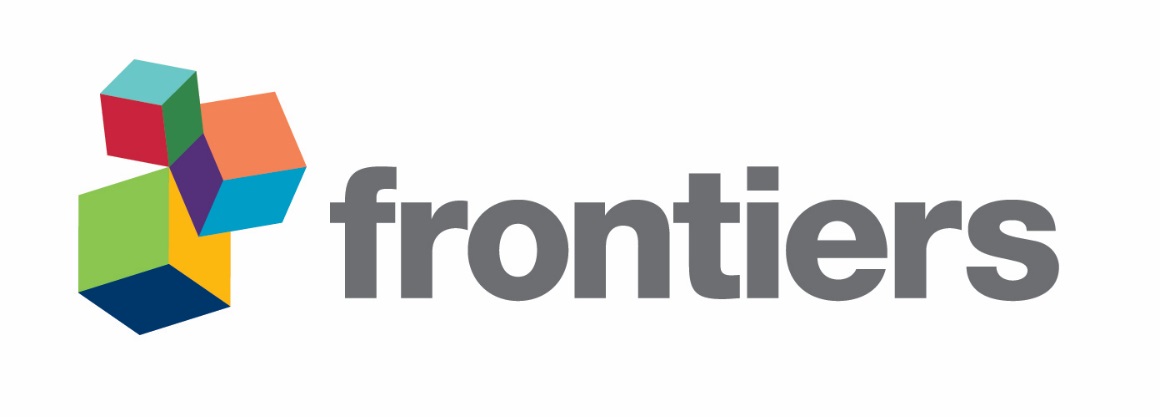
**
