# Supplementary material for: Longitudinal changes in oral conditions and oral candidiasis in palliative care inpatients: a longitudinal observational study
Source: Front Dent Med. 2026 Jul 2;7:1831411. doi: 10.3389/fdmed.2026.1831411 (PMC13372980; doi:10.3389/fdmed.2026.1831411)
Supplement: Supplementary file 2 [file Datasheet1.pdf]

Table S1. Oral condition at first visit (n = 300)

No.	(1) Age	(2) Sex	(3) Disease site	(4) Chief Complaint	(5) Outcome	Oral candidiasis		OHAT (Before)									OHAT (After)								
						Before	After	Lips	Tongue	Gums	Saliva	Natural teeth	Dentures	Oral cleanliness	Dental pain	Total score	Lips	Tongue	Gums	Saliva	Natural teeth	Dentures	Oral cleanliness	Dental pain	Total score
1	30	M	Lung cancer	Oral care	Death	Impossible	Impossible	1	0	0	0	1	0	1	0	3	0	0	0	2	0	2	0	0	4
2	42	M	Pancreas	Oral care	Death	Impossible	-	0	0	0	0	0	0	1	0	1	-	-	-	-	-	-	-	-	-
3	44	F	Head and Neck	Oral care	Discharge	Impossible	Impossible	0	0	0	0	0	0	0	0	0	0	0	0	0	0	0	0	0	0
4	45	F	Uterus, Ovaries	Adrenal cortical steroids	Death	Impossible	Impossible	1	1	0	0	0	0	0	0	2	1	1	1	1	0	0	0	0	4
5	45	M	Hepatocellular carcinoma	Adrenal cortical steroids	Death	Impossible	-	2	0	1	1	0	0	0	0	4	-	-	-	-	-	-	-	-	-
6	46	F	Uterus, Ovaries	Oral care	Death	Impossible	Impossible	0	1	0	0	1	0	0	0	2	0	1	0	0	1	0	0	0	2
7	46	F	Large intestine	Oral care	Death	Impossible	-	1	0	0	1	0	0	1	0	3	-	-	-	-	-	-	-	-	-
8	46	M	Stomach	Adrenal cortical steroids	Discharge	Impossible	-	0	0	0	0	0	0	0	0	0	-	-	-	-	-	-	-	-	-
9	47	F	Uterus, Ovaries	Oral care	Discharge	Impossible	Impossible	1	1	1	1	0	0	0	0	4	0	0	0	0	0	0	0	0	0
10	47	F	Large intestine	Oral care	Discharge	Impossible	Impossible	1	1	1	1	1	0	1	0	6	0	1	1	1	1	0	1	0	5
11	48	F	Breast	Oral candidiasis	Discharge	Possible	-	0	0	0	0	0	0	0	0	0	-	-	-	-	-	-	-	-	-
12	49	F	Head and Neck	Oral care	Discharge	Impossible	Impossible	0	0	0	0	0	0	0	0	0	0	0	0	0	0	0	0	0	0
13	49	M	Urology	Osteoclastic inhibitor	Discharge	Impossible	Impossible	0	0	0	0	0	0	0	0	0	0	1	0	0	0	0	0	0	1
14	49	F	Uterus, Ovaries	Oral care	Discharge	Impossible	Impossible	0	0	0	0	0	0	0	0	0	1	1	0	1	0	0	0	0	3
15	49	F	Uterus, Ovaries	Xerostomia	Discharge	Impossible	Impossible	1	1	1	1	0	0	1	0	5	1	1	1	1	0	0	0	0	4
16	49	F	Large intestine	Adrenal cortical steroids	Death	Impossible	-	2	1	1	1	0	0	0	0	5	-	-	-	-	-	-	-	-	-
17	50	M	Pancreas	Oral care	Discharge	Impossible	Impossible	0	0	0	0	0	0	0	0	0	0	0	0	0	0	0	0	0	0

18	50	F	Pancreas	Xerostomia	Discharge	Impossible	Impossible	0	1	0	0	0	0	1	0	2	0	0	0	0	0	0	1	0	1
19	50	M	Large intestine	Osteoclastic inhibitor	Death	Impossible	Impossible	0	1	0	0	1	0	1	0	3	0	1	0	1	1	0	0	0	3
20	50	M	Urology	Oral care	Death	Impossible	Impossible	1	1	0	0	0	0	0	0	2	1	1	0	1	0	0	1	0	4
21	51	F	Bile duct, Gallbladder	Pain	Discharge	Impossible	Impossible	1	0	0	0	0	0	0	0	1	1	0	0	0	0	0	0	0	1
22	52	F	Breast	Oral care	Death	Impossible	-	2	0	1	0	0	0	1	0	4	-	-	-	-	-	-	-	-	-
23	52	M	Pancreas	Oral care	Death	Impossible	-	1	0	0	1	0	0	1	0	3	-	-	-	-	-	-	-	-	-
24	53	M	Stomach	Pain	Death	Impossible	Impossible	0	0	0	0	2	0	0	0	2	0	1	1	1	2	0	0	0	5
25	54	M	Lung	Oral care	Discharge	Impossible	Impossible	0	0	0	0	0	0	1	0	1	0	0	0	0	2	0	1	0	3
26	55	M	Urology	Pain	Discharge	Impossible	Impossible	0	0	0	0	1	0	0	0	1	0	0	0	0	1	0	0	0	1
27	55	F	Uterus, Ovaries	Osteoclastic inhibitor	Discharge	Impossible	-	0	1	0	0	0	0	0	0	1	-	-	-	-	-	-	-	-	-
28	56	F	Lung	Oral care	Discharge	Impossible	Impossible	0	0	0	0	0	0	0	0	0	0	0	0	0	0	0	0	0	0
29	56	M	Urology	Chemotherapy	Discharge	Impossible	Impossible	0	0	0	0	1	0	0	0	1	0	0	0	0	0	0	0	0	0
30	56	F	Uterus, Ovaries	Adrenal cortical steroids	Discharge	Impossible	Impossible	0	1	0	0	0	0	1	0	2	0	1	0	0	0	0	0	0	1
31	56	M	Large intestine	Adrenal cortical steroids	Discharge	Impossible	Impossible	0	0	0	0	0	0	0	0	0	0	1	0	0	0	0	1	0	2
32	56	M	Pancreas	Oral care	Death	Impossible	Impossible	0	1	0	0	0	0	0	0	1	0	1	0	0	0	0	2	0	3
33	56	M	Malignant lymphoma	Osteoclastic inhibitor	Death	Impossible	Impossible	1	0	1	1	0	0	2	0	5	1	0	1	1	0	0	3	0	6
34	56	M	Lung	Oral care	Discharge	Impossible	-	1	0	1	1	0	0	1	0	4	-	-	-	-	-	-	-	-	-
35	57	M	Hepatocellular carcinoma	Xerostomia	Discharge	Possible	Impossible	0	1	0	1	0	0	1	0	3	0	1	0	1	0	0	1	0	3
36	57	F	Lung	Oral care	Discharge	Impossible	-	0	0	0	0	0	0	0	0	0	-	-	-	-	-	-	-	-	-
37	57	M	Pancreas	Adrenal cortical steroids	Discharge	Impossible	-	0	0	0	0	0	0	0	0	0	-	-	-	-	-	-	-	-	-

38	58	M	Pancreas	Taste disorder	Discharge	Impossible	Impossible	0	2	0	0	0	0	1	0	3	0	0	0	0	0	0	0	0	0
39	58	M	Pancreas	Adrenal cortical steroids	Discharge	Impossible	Impossible	0	1	1	1	0	0	1	0	4	0	1	0	0	0	0	0	0	1
40	59	M	Stomach	Chemotherapy	Discharge	Impossible	Impossible	0	0	0	0	0	0	1	0	1	0	0	0	0	0	0	0	0	0
41	59	F	Uterus, Ovaries	Xerostomia	Discharge	Impossible	Impossible	0	0	0	1	0	0	0	0	1	0	1	0	1	0	0	0	0	2
42	59	M	Bile duct, Gallbladder	Taste disorder	Discharge	Impossible	Impossible	1	1	1	1	0	0	1	0	5	1	1	1	1	0	0	1	0	5
43	60	F	Uterus, Ovaries	Chemotherapy	Discharge	Impossible	Impossible	0	1	0	0	0	0	0	0	1	0	0	0	0	0	0	0	0	0
44	60	M	Lung	Adrenal cortical steroids	Death	Impossible	Impossible	0	0	0	0	0	0	2	0	2	0	0	0	0	0	0	1	0	1
45	60	M	Stomach	Pain	Death	Impossible	Impossible	0	0	0	0	0	0	0	0	0	0	0	0	1	0	0	0	0	1
46	60	M	Large intestine	Oral care	Discharge	Impossible	Impossible	0	1	0	0	0	0	1	0	2	0	0	1	1	0	0	1	0	3
47	60	M	Lung	Osteoclastic inhibitor	Discharge	Impossible	-	0	1	0	0	0	0	1	0	2	-	-	-	-	-	-	-	-	-
48	60	F	Pancreas	Chemotherapy	Discharge	Impossible	-	0	0	0	0	0	0	0	0	0	-	-	-	-	-	-	-	-	-
49	61	F	Pancreas	Xerostomia	Death	Impossible	Impossible	1	0	1	1	0	0	0	0	3	1	0	1	1	0	0	0	0	0
50	61	M	Large intestine	Oral care	Death	Impossible	Impossible	0	1	1	1	0	0	0	0	3	0	1	1	1	0	0	0	0	0
51	61	F	Pancreas	Adrenal cortical steroids	Death	Impossible	Impossible	0	1	0	0	0	0	0	1	2	0	1	0	0	0	0	0	1	2
52	61	M	Large intestine	Adrenal cortical steroids	Death	Impossible	Impossible	0	0	0	0	0	0	0	0	0	0	1	0	0	1	0	1	0	3
53	61	M	Urology	Oral care	Death	Impossible	-	0	1	1	2	1	0	1	0	6	-	-	-	-	-	-	-	-	-
54	61	M	Large intestine	Chemotherapy	Death	Impossible	-	0	1	1	1	0	0	0	0	3	-	-	-	-	-	-	-	-	-
55	61	F	Pancreas	Adrenal cortical steroids	Discharge	Impossible	-	0	0	0	0	0	0	0	0	0	-	-	-	-	-	-	-	-	-
56	62	F	Hepatocellular carcinoma	Pain	Death	Impossible	Impossible	0	0	0	0	1	1	0	1	3	0	0	0	0	0	0	0	0	0
57	62	F	Colorectal malignant polyps S/O	Adrenal cortical steroids	Discharge	Impossible	Impossible	0	1	0	0	0	0	0	0	1	0	1	0	0	0	0	0	0	1

58	62	M	Large intestine	Osteoclastic inhibitor	Discharge	Impossible	Impossible	0	0	0	-	0	0	0	0	0	0	1	1	1	0	0	0	0	3
59	63	M	Lung	Chemotherapy	Discharge	Impossible	Impossible	0	0	0	0	0	0	0	0	0	0	0	0	0	0	0	0	0	0
60	63	M	Stomach	Oral care	Discharge	Impossible	Impossible	0	1	0	0	0	0	2	0	3	0	0	0	0	0	0	1	0	1
61	63	M	Pancreas	Xerostomia	Death	Impossible	Impossible	0	1	1	1	2	0	1	0	6	0	1	1	1	2	0	1	0	6
62	63	M	Stomach	Pain	Discharge	Impossible	-	1	0	0	0	0	0	1	0	2	-	-	-	-	-	-	-	-	-
63	63	M	Bile duct, Gallbladder	Xerostomia	Discharge	Possible	-	1	0	0	0	0	0	0	0	1	-	-	-	-	-	-	-	-	-
64	64	M	Lung	Oral care	Discharge	Impossible	Impossible	0	0	0	1	0	0	0	0	1	0	0	0	0	0	0	0	0	0
65	64	M	Lung	Oral care	Discharge	Impossible	Impossible	0	0	0	0	1	0	0	0	1	0	0	0	0	1	0	0	0	1
66	64	M	Lung	Adrenal cortical steroids	Discharge	Impossible	Impossible	0	1	0	1	0	0	1	0	3	0	1	0	0	0	0	0	0	1
67	64	M	Pancreas	Adrenal cortical steroids	Death	Impossible	Impossible	0	0	0	1	0	0	0	0	1	0	0	0	2	0	0	0	0	2
68	64	M	Head and Neck	Speech disorder	Death	Impossible	Impossible	1	1	1	1	0	0	0	0	4	0	1	1	1	0	0	0	0	3
69	64	M	Large intestine	Denture misfit	Death	Impossible	Possible	0	0	0	0	0	2	2	0	4	0	1	0	1	1	0	1	0	4
70	64	M	Head and Neck	Oral care	Discharge	Impossible	Impossible	0	1	2	1	1	2	2	0	9	0	1	2	1	1	2	2	0	9
71	64	M	Lung	Adrenal cortical steroids	Discharge	Impossible	-	0	1	0	1	0	0	1	0	3	-	-	-	-	-	-	-	-	-
72	65	F	Stomach	Oral care	Death	Impossible	Impossible	0	1	0	0	0	0	1	0	2	0	1	0	0	0	0	0	0	1
73	65	F	Locally advanced gastric cancer	Osteoclastic inhibitor	Death	Impossible	Impossible	0	0	0	0	0	0	0	0	0	2	1	0	0	0	0	0	0	3
74	65	M	Esophagus	Oral care	Discharge	Impossible	Possible	0	1	1	1	0	0	0	0	3	0	1	1	1	0	0	0	0	3
75	65	F	Uterus, Ovaries	Xerostomia	Discharge	Impossible	Impossible	1	0	0	0	0	0	0	0	1	1	0	1	1	0	0	1	0	4
76	65	M	Hepatocellular carcinoma	Oral care	Death	Impossible	Impossible	1	0	1	0	2	0	2	0	6	0	1	2	2	2	0	1	0	8
77	65	M	Lung	Adrenal cortical steroids	Death	Impossible	-	0	0	0	0	0	0	0	0	0	-	-	-	-	-	-	-	-	-

78	65	M	Lung	Oral care	Discharge	Impossible	-	0	0	0	0	1	0	0	0	1	-	-	-	-	-	-	-	-	-
79	66	F	Breast	Oral care	Discharge	Impossible	Possible	0	0	0	0	0	0	0	0	0	0	0	0	0	0	0	0	0	0
80	66	M	Lung	Oral candidiasis	Discharge	Impossible	Impossible	0	2	2	0	0	0	1	0	5	0	0	0	0	0	0	0	0	0
81	66	M	Stomach	Adrenal cortical steroids	Death	Impossible	Impossible	0	0	0	0	1	0	0	0	1	0	0	0	0	0	0	1	0	1
82	66	F	Lung	Chemotherapy	Discharge	Impossible	Impossible	0	0	0	0	1	0	0	0	1	0	0	0	0	1	0	0	0	1
83	66	M	Bile duct, Gallbladder	Oral care	Discharge	Impossible	Impossible	0	1	0	1	0	0	1	0	3	0	1	0	0	0	0	0	0	1
84	66	M	Lung	Xerostomia	Death	Impossible	Impossible	0	1	1	1	0	0	0	0	3	1	1	1	1	0	0	0	0	4
85	66	M	Bile duct, Gallbladder	Oral care	Discharge	Impossible	Impossible	0	1	0	0	0	0	1	0	2	0	1	1	1	0	0	1	0	4
86	66	M	Esophagus	Oral care	Discharge	Impossible	Impossible	1	1	1	2	0	0	1	0	6	1	1	1	2	0	0	1	0	6
87	66	M	Bile duct, Gallbladder	Oral care	Discharge	Impossible	-	0	0	0	0	0	0	1	0	1	-	-	-	-	-	-	-	-	-
88	67	F	Uterus, Ovaries	Oral care	Discharge	Impossible	Impossible	1	0	0	0	0	0	0	0	1	0	0	0	0	0	0	0	0	0
89	67	F	Hepatocellular carcinoma	Osteoclastic inhibitor	Discharge	Impossible	Impossible	0	0	0	0	0	0	0	0	0	0	0	0	0	0	0	0	0	0
90	67	M	Head and Neck	Adrenal cortical steroids	Death	Possible	Impossible	0	1	1	0	1	0	1	0	4	0	0	1	0	0	0	0	0	1
91	67	F	Pancreas	Oral care	Discharge	Impossible	Possible	0	0	0	1	0	0	2	0	3	0	0	0	1	0	0	0	0	1
92	67	M	Stomach	Osteoclastic inhibitor	Death	Impossible	Impossible	0	0	0	0	0	0	0	1	1	0	1	1	1	0	0	0	0	3
93	67	M	Malignant lymphoma	Oral care	Death	Impossible	Impossible	1	1	0	0	0	0	0	0	2	0	1	1	1	0	0	1	0	4
94	67	M	Stomach	Adrenal cortical steroids	Death	Possible	Impossible	0	0	1	1	0	0	2	0	4	0	0	1	1	0	0	2	0	4
95	67	M	Large intestine	Chemotherapy	Discharge	Possible	Impossible	0	1	1	1	2	0	2	0	7	0	1	0	0	2	0	1	0	4
96	67	F	Large intestine	Adrenal cortical steroids	Discharge	Impossible	Impossible	0	0	0	0	0	0	0	0	0	1	1	1	1	0	0	0	0	4
97	67	M	Lung	Oral care	Discharge	Impossible	Impossible	0	1	1	1	1	0	1	0	5	0	1	1	1	1	0	1	0	5

98	67	M	Malignant lymphoma	Oral care	Discharge	Impossible	Impossible	1	0	2	1	2	0	2	2	10	1	0	2	1	2	0	1	0	7
99	67	F	Breast	Adrenal cortical steroids	Discharge	Possible	-	1	1	0	0	0	0	1	0	3	-	-	-	-	-	-	-	-	-
100	68	F	Peritoneal carcinoma	Oral care	Discharge	Impossible	Impossible	0	0	0	0	0	0	0	0	0	0	0	0	0	0	0	0	0	0
101	68	M	Urology	Taste disorder	Discharge	Impossible	Impossible	1	0	0	2	0	0	0	2	5	0	1	0	0	0	0	1	0	2
102	68	F	Unknown primary origin	Adrenal cortical steroids	Discharge	Impossible	Impossible	0	1	0	0	0	0	2	0	3	0	1	0	0	0	0	1	0	2
103	68	F	Peritoneal carcinoma	Oral care	Death	Impossible	Impossible	0	0	0	0	1	0	0	0	1	0	0	1	1	1	0	0	0	3
104	68	M	Stomach	Adrenal cortical steroids	Discharge	Impossible	Impossible	0	1	0	0	1	0	1	0	3	0	1	0	0	1	0	1	0	3
105	68	M	Stomach	Oral care	Discharge	Possible	Impossible	0	2	2	1	0	0	2	1	8	0	0	1	1	0	0	1	0	3
106	68	M	Urology	Osteoclastic inhibitor	Death	Impossible	Impossible	0	1	0	0	1	0	1	0	3	0	1	1	1	1	0	0	0	4
107	68	M	Urology	Oral care	Death	Possible	-	0	1	1	1	0	1	0	0	4	-	-	-	-	-	-	-	-	-
108	69	M	Stomach	Adrenal cortical steroids	Death	Impossible	Impossible	0	0	0	0	1	0	0	0	1	0	0	0	0	0	0	2	0	2
109	69	F	Large intestine	Denture misfit	Discharge	Impossible	Impossible	0	0	0	0	0	2	0	0	2	0	0	0	0	0	0	2	0	2
110	69	F	Unknown primary origin	Oral care	Discharge	Impossible	Impossible	1	0	0	0	1	0	2	0	4	1	0	0	0	1	0	1	0	3
111	69	F	Uterine sarcoma	Tooth	Discharge	Impossible	Impossible	2	1	2	0	0	0	1	1	7	1	1	1	1	0	0	1	0	5
112	69	F	Breast	Xerostomia	Discharge	Impossible	-	0	0	0	0	0	0	2	0	2	-	-	-	-	-	-	-	-	-
113	69	F	Large intestine	Osteoclastic inhibitor	Discharge	Impossible	-	0	0	0	0	0	0	0	0	0	-	-	-	-	-	-	-	-	-
114	70	M	Hepatocellular carcinoma	Oral care	Discharge	Impossible	Impossible	0	0	0	0	0	0	0	0	0	0	0	0	0	0	0	0	0	0
115	70	M	Esophagus	Oral candidiasis	Discharge	Possible	Impossible	0	0	0	0	0	0	1	0	1	0	0	0	0	0	0	0	0	0
116	70	M	Malignant Brain Tumors	Adrenal cortical steroids	Death	Impossible	Impossible	1	0	0	0	0	0	0	0	1	1	0	0	0	0	0	0	0	1
117	70	F	Large intestine	Oral care	Death	Impossible	Impossible	0	0	0	0	0	0	0	0	0	0	0	0	1	0	0	0	0	1

118	70	F	Uterus, Ovaries	Oral care	Discharge	Impossible	Impossible	0	1	0	0	0	0	1	0	2	0	0	0	0	0	0	1	0	1
119	70	M	Urology	Oral care	Discharge	Impossible	Impossible	0	1	0	0	0	0	0	0	1	0	1	0	0	0	0	0	0	1
120	70	F	Hepatocellular carcinoma	Adrenal cortical steroids	Discharge	Impossible	Impossible	0	0	0	0	0	0	1	0	1	0	0	0	0	1	0	1	0	2
121	70	F	Pancreas	Adrenal cortical steroids	Discharge	Impossible	Impossible	1	1	0	2	0	0	1	0	5	0	0	1	0	0	1	0	0	2
122	70	M	Bile duct, Gallbladder	Xerostomia	Discharge	Impossible	Impossible	1	0	0	0	0	0	1	0	2	0	0	1	1	0	0	1	0	3
123	70	M	Stomach	Adrenal cortical steroids	Discharge	Impossible	Impossible	0	1	0	0	2	0	1	0	4	0	1	0	0	2	1	1	0	5
124	70	F	Esophagus	Adrenal cortical steroids	Death	Impossible	Impossible	1	1	1	1	1	0	1	0	6	1	1	1	1	1	0	1	0	6
125	70	F	Lung	Taste disorder	Discharge	Impossible	-	0	0	0	0	0	0	0	1	1	-	-	-	-	-	-	-	-	-
126	70	F	Lung	Adrenal cortical steroids	Discharge	Impossible	-	0	0	0	0	0	0	1	0	1	-	-	-	-	-	-	-	-	-
127	71	M	Urology	Osteoclastic inhibitor	Death	Impossible	Impossible	0	0	0	1	0	0	1	0	2	0	0	0	0	0	0	0	0	0
128	71	M	Large intestine	Oral bleeding	Death	Impossible	Impossible	1	1	1	1	0	0	0	0	4	1	2	1	1	0	0	0	0	0
129	71	F	Large intestine	Denture misfit	Discharge	Impossible	Impossible	0	0	2	0	0	0	1	0	3	0	0	0	0	0	0	0	0	0
130	71	M	Urology	Oral care	Death	Impossible	Impossible	0	0	0	0	0	0	1	0	1	0	0	1	0	0	0	1	0	2
131	71	M	Stomach	Adrenal cortical steroids	Discharge	Impossible	Impossible	0	0	0	0	0	2	0	0	2	0	0	0	0	0	2	0	0	2
132	71	M	Head and Neck	Tooth	Death	Impossible	Impossible	0	1	1	1	0	0	1	0	4	0	1	1	1	0	0	0	0	3
133	71	M	Pancreas	Xerostomia	Discharge	Possible	Possible	1	1	1	1	0	0	2	0	6	0	0	1	1	0	0	1	0	3
134	71	M	Large intestine	Pain	Death	Possible	Impossible	2	1	2	1	0	0	1	0	7	1	2	2	0	0	0	1	0	6
135	71	F	Urology	Taste disorder	Death	Possible	-	1	1	0	0	0	0	1	1	4	-	-	-	-	-	-	-	-	-
136	71	M	Esophagus	Oral candidiasis	Discharge	Impossible	-	0	0	1	1	0	0	2	0	4	-	-	-	-	-	-	-	-	-
137	72	M	Large intestine	Oral care	Death	Impossible	Impossible	0	0	1	1	0	0	1	0	3	0	0	0	0	0	0	0	0	0

138	72	M	Esophagus	Oral care	Discharge	Impossible	Impossible	0	1	0	0	0	1	1	0	3	0	1	0	0	0	0	0	0	1
139	72	M	Large intestine	Oral care	Death	Impossible	Impossible	0	1	0	0	0	0	1	0	2	0	0	1	1	0	0	0	0	2
140	72	M	Esophagus	Oral candidiasis	Discharge	Impossible	Impossible	0	1	0	0	0	0	1	0	2	0	1	0	0	0	0	1	0	2
141	72	F	Pancreas	Oral care	Death	Impossible	Impossible	0	1	1	1	0	0	0	0	3	0	1	1	1	0	0	0	0	3
142	72	F	Malignant Brain Tumors	Adrenal cortical steroids	Discharge	Impossible	Possible	1	0	1	1	0	0	0	1	4	1	0	1	0	0	1	0	0	3
143	72	F	Pancreas	Oral care	Death	Impossible	Impossible	0	0	0	0	1	2	1	0	4	0	0	0	0	1	2	1	0	4
144	72	F	Stomach	Adrenal cortical steroids	Discharge	Impossible	Impossible	0	1	1	1	0	0	1	0	4	0	1	1	1	0	0	1	0	4
145	72	M	Large intestine	Adrenal cortical steroids	Discharge	Impossible	Impossible	0	0	0	0	0	0	0	0	0	1	1	1	1	0	0	1	0	5
146	72	M	Urology	Osteoclastic inhibitor	Discharge	Impossible	Possible	1	1	1	1	0	0	1	0	5	0	1	1	1	0	0	2	0	5
147	72	M	Head and Neck	Pain	Death	Impossible	Impossible	1	0	2	0	0	0	0	0	3	1	0	1	1	0	2	0	2	7
148	72	M	Unknown primary origin	Adrenal cortical steroids	Death	Impossible	-	1	0	1	1	0	0	1	0	4	-	-	-	-	-	-	-	-	-
149	72	M	Large intestine	Denture misfit	Discharge	Impossible	-	0	0	0	0	0	1	0	0	1	-	-	-	-	-	-	-	-	-
150	72	F	Lung	Chemotherapy	Discharge	Impossible	-	0	0	0	0	0	0	0	0	0	-	-	-	-	-	-	-	-	-
151	72	M	Lung	Oral bleeding	Discharge	Impossible	-	0	1	2	1	1	0	2	0	7	-	-	-	-	-	-	-	-	-
152	73	F	Lung	Taste disorder	Discharge	Possible	Impossible	0	0	0	0	0	0	0	0	0	0	0	0	0	0	0	0	0	0
153	73	F	Peritoneal carcinoma	Oral care	Discharge	Impossible	Impossible	0	0	0	0	0	0	0	0	0	0	0	0	0	0	0	0	0	0
154	73	M	Small intestine	Oral care	Discharge	Possible	Possible	0	0	0	0	0	0	0	0	0	0	0	0	0	0	0	0	0	0
155	73	F	Stomach	Oral care	Discharge	Impossible	Impossible	0	0	0	0	0	0	1	0	1	0	0	0	0	0	0	0	0	0
156	73	M	Pancreas	Adrenal cortical steroids	Death	Possible	Possible	0	0	0	0	1	0	2	0	3	0	0	0	1	0	0	1	0	2
157	73	M	Lung	Adrenal cortical steroids	Discharge	Impossible	Impossible	0	0	1	1	0	0	0	0	2	0	0	1	1	0	0	0	0	2

158	73	M	Stomach	Oral care	Discharge	Impossible	Impossible	0	0	0	0	1	0	1	0	2	0	0	0	0	1	0	1	0	2
159	73	F	Pancreas	Adrenal cortical steroids	Discharge	Impossible	Impossible	0	0	0	0	2	0	1	0	3	0	0	0	0	2	0	1	0	3
160	73	F	Uterus, Ovaries	Pain	Death	Possible	Impossible	0	0	0	0	1	0	0	0	1	0	1	0	0	1	0	2	0	4
161	73	F	Lung	Oral care	Death	Impossible	Impossible	0	0	1	1	0	0	1	0	3	0	1	1	1	0	0	1	0	4
162	73	M	Urology	Oral care	Discharge	Impossible	Impossible	0	0	0	0	0	0	0	0	0	0	1	1	1	0	0	1	0	4
163	73	M	Lung	Oral candidiasis	Death	Possible	Impossible	1	1	0	0	0	0	0	0	2	1	1	1	1	0	0	1	0	5
164	73	M	Urology	Osteoclastic inhibitor	Death	Possible	Impossible	1	1	1	1	0	0	2	0	6	0	1	1	2	0	0	1	0	5
165	73	M	Head and Neck	Oral care	Death	Impossible	-	0	1	1	2	1	0	2	0	7	-	-	-	-	-	-	-	-	-
166	73	F	Large intestine	Oral bleeding	Discharge	Impossible	-	0	0	0	0	0	0	0	0	0	-	-	-	-	-	-	-	-	-
167	74	F	Pancreas	Adrenal cortical steroids	Discharge	Impossible	Impossible	0	0	0	0	0	0	0	0	0	0	0	0	0	0	0	0	0	0
168	74	F	Pancreas	Adrenal cortical steroids	Discharge	Possible	Impossible	0	0	0	0	0	0	0	0	0	0	0	0	0	0	0	0	0	0
169	74	F	Lung	Adrenal cortical steroids	Discharge	Possible	Impossible	1	0	0	0	0	0	0	0	1	1	0	0	0	0	0	0	0	1
170	74	F	Pancreas	Tooth	Death	Impossible	Impossible	0	1	0	0	1	0	2	0	4	0	1	0	1	0	1	0	0	3
171	74	M	Lung	Oral care	Death	Impossible	Impossible	0	1	1	1	0	0	2	0	5	0	1	1	1	0	0	1	0	4
172	74	F	Pancreas	Oral care	Death	Impossible	Impossible	1	1	1	1	1	0	1	1	7	0	1	0	1	1	0	1	0	4
173	74	F	Large intestine	Adrenal cortical steroids	Discharge	Impossible	Impossible	1	1	1	1	0	1	1	0	6	0	1	1	2	0	1	1	0	6
174	74	F	Pancreas	Chemotherapy 前	Death	Impossible	-	0	0	0	0	0	0	0	0	0	-	-	-	-	-	-	-	-	-
175	74	M	Pancreas	Adrenal cortical steroids	Death	Impossible	-	0	1	1	1	0	0	1	0	4	-	-	-	-	-	-	-	-	-
176	74	M	Head and Neck	Xerostomia	Death	Impossible	-	1	1	1	1	0	0	1	0	5	-	-	-	-	-	-	-	-	-
177	74	F	Breast	Taste disorder	Discharge	Impossible	-	0	0	0	0	0	0	0	0	0	-	-	-	-	-	-	-	-	-

178	75	M	Urology	Osteoclastic inhibitor	Discharge	Impossible	Impossible	0	1	0	0	0	0	1	0	2	0	0	0	0	0	0	0	0	0
179	75	F	Peritoneal carcinoma S/O	Oral care	Discharge	Impossible	Impossible	0	0	0	0	0	0	0	0	0	0	0	0	0	0	0	0	0	0
180	75	F	Urology	Adrenal cortical steroids	Death	Impossible	Impossible	0	1	0	0	0	0	0	0	1	0	1	0	0	0	0	0	0	1
181	75	M	Malignant lymphoma	Oral care	Death	Impossible	Impossible	0	1	1	0	0	0	0	0	2	0	1	1	0	0	0	0	0	2
182	75	M	Pancreas	Taste disorder	Discharge	Impossible	Impossible	0	1	0	0	0	0	1	0	2	0	1	0	0	0	0	1	0	2
183	75	F	Malignant lymphoma	Oral care	Death	Impossible	Impossible	0	1	1	1	0	0	0	1	4	0	1	1	1	0	0	0	0	3
184	75	F	Large intestine	Oral care	Death	Impossible	Impossible	0	0	0	0	0	0	0	0	0	0	1	1	1	0	0	0	0	3
185	75	M	Pancreas	Oral care	Death	Impossible	Impossible	1	1	0	1	0	0	1	0	4	1	1	0	1	0	0	1	0	4
186	75	M	Head and Neck	Pain	Discharge	Possible	Impossible	0	0	0	0	0	0	0	0	0	0	1	1	1	0	0	1	0	4
187	75	M	Vulva Cancer	Adrenal cortical steroids	Discharge	Impossible	-	0	1	0	0	0	0	0	0	1	-	-	-	-	-	-	-	-	-
188	75	M	Pancreas	Adrenal cortical steroids	Discharge	Impossible	-	0	1	1	1	0	0	0	0	3	-	-	-	-	-	-	-	-	-
189	75	M	Large intestine	Adrenal cortical steroids	Discharge	Impossible	-	0	1	0	0	0	0	1	0	2	-	-	-	-	-	-	-	-	-
190	75	M	Lung	Oral care	Discharge	Impossible	-	0	1	0	1	0	0	1	0	3	-	-	-	-	-	-	-	-	-
191	76	F	Malignant soft tissue tumor of the neck	Adrenal cortical steroids	Discharge	Impossible	Impossible	0	1	0	0	0	0	0	0	1	0	0	0	0	0	0	0	0	0
192	76	M	Esophagus	Radiation therapy	Discharge	Impossible	Impossible	0	1	0	1	0	0	0	0	2	0	0	0	0	0	0	0	0	0
193	76	F	Head and Neck	Oral care	Discharge	Impossible	Impossible	0	1	0	0	2	1	2	0	6	0	0	0	0	0	0	0	0	0
194	76	M	Large intestine	Oral care	Death	Impossible	Impossible	0	0	0	0	0	0	1	0	1	0	0	0	0	0	0	1	0	1
195	76	F	Lung	Oral care	Discharge	Impossible	Impossible	0	1	0	0	1	0	1	0	3	0	0	0	0	1	0	1	0	2
196	76	M	Lung	Adrenal cortical steroids	Death	Impossible	Impossible	1	0	1	1	0	0	0	0	3	1	0	1	1	0	0	0	0	3
197	76	M	Pancreas	Xerostomia	Death	Impossible	-	1	1	0	0	0	0	0	0	2	-	-	-	-	-	-	-	-	-

198	76	M	Stomach	Adrenal cortical steroids	Discharge	Impossible	-	0	1	0	0	0	0	1	0	2	-	-	-	-	-	-	-	-	-
199	76	F	Pancreas	Denture misfit	Discharge	Impossible	-	0	0	1	0	1	1	0	1	4	-	-	-	-	-	-	-	-	-
200	76	M	Head and Neck	Oral care	Discharge	Impossible	-	0	0	0	0	0	0	0	0	0	-	-	-	-	-	-	-	-	-
201	77	F	Urology	Taste disorder	Discharge	Impossible	Impossible	0	0	0	0	0	0	0	0	0	0	0	0	0	0	0	0	0	0
202	77	M	Large intestine	Adrenal cortical steroids	Discharge	Impossible	Impossible	0	0	0	0	0	0	1	0	1	0	0	0	0	0	0	0	0	0
203	77	M	Thymic carcinoma	Oral care	Discharge	Impossible	Impossible	0	1	0	0	0	0	1	0	2	0	1	0	0	0	0	1	0	2
204	77	M	Stomach	Oral candidiasis	Discharge	Possible	Impossible	0	0	0	0	0	2	0	0	2	0	0	0	0	0	2	0	0	2
205	77	M	Head and Neck	Adrenal cortical steroids	Death	Impossible	Impossible	0	0	1	1	1	0	0	0	3	0	0	0	1	1	0	2	0	4
206	77	M	Pancreas	Taste disorder	Discharge	Impossible	Impossible	0	1	1	1	0	0	1	0	4	0	1	1	1	0	0	1	0	4
207	77	M	Lung	Adrenal cortical steroids	Discharge	Impossible	Impossible	0	1	1	1	0	0	1	0	4	0	1	1	1	0	0	1	0	4
208	77	M	Urology	Oral care	Discharge	Impossible	Impossible	0	0	1	0	0	0	2	0	3	1	1	1	0	0	0	1	0	4
209	77	M	Esophagus	Oral care	Death	Impossible	Impossible	0	1	0	0	2	0	2	0	5	1	1	0	0	2	0	2	2	8
210	77	F	Uterus, Ovaries	Oral care	Death	Impossible	-	0	0	0	0	0	0	2	0	2	-	-	-	-	-	-	-	-	-
211	77	F	Head and Neck	Adrenal cortical steroids	Death	Impossible	-	0	0	0	0	0	0	0	0	0	-	-	-	-	-	-	-	-	-
212	77	F	Lung	Xerostomia	Discharge	Impossible	-	1	0	0	0	0	0	0	0	1	-	-	-	-	-	-	-	-	-
213	77	M	Urology	Adrenal cortical steroids	Discharge	Impossible	-	0	1	0	0	0	0	0	0	1	-	-	-	-	-	-	-	-	-
214	78	F	Lung	Oral care	Discharge	Impossible	Impossible	2	0	0	0	0	0	0	0	2	0	0	0	0	0	0	0	0	0
215	78	F	Uterus, Ovaries	Oral care	Death	Impossible	Impossible	1	0	1	1	0	0	1	0	4	0	0	0	1	0	0	0	0	1
216	78	M	Small intestine	Adrenal cortical steroids	Death	Impossible	Impossible	0	0	0	0	0	0	0	0	0	0	0	0	0	0	0	1	0	1
217	78	M	Hepatocellular carcinoma	Taste disorder	Discharge	Impossible	Possible	0	0	0	0	0	0	1	0	1	0	0	0	0	0	0	1	0	1

218	78	M	Pancreas	Adrenal cortical steroids	Death	Possible	Impossible	1	1	1	1	0	0	1	0	5	1	1	1	1	0	0	1	0	5
219	78	M	Bile duct, Gallbladder	Oral care	Discharge	Impossible	Impossible	0	0	1	1	2	2	1	0	7	0	0	1	1	2	2	0	0	6
220	78	M	Urology	Taste disorder	Discharge	Impossible	-	0	0	0	0	0	0	0	0	0	-	-	-	-	-	-	-	-	-
221	78	M	Urology	Oral candidiasis	Discharge	Impossible	-	0	1	0	0	0	0	0	0	1	-	-	-	-	-	-	-	-	-
222	79	M	Urology	Osteoclastic inhibitor	Discharge	Impossible	Impossible	0	0	0	0	0	0	0	0	0	0	0	0	0	0	0	0	0	0
223	79	F	Unknown primary origin	Radiation therapy	Discharge	Impossible	Impossible	0	0	0	0	0	0	0	0	0	0	0	0	0	0	0	0	0	0
224	79	M	Pancreas	Adrenal cortical steroids	Discharge	Impossible	Possible	0	0	0	0	0	0	1	0	1	0	1	1	1	0	0	1	0	4
225	79	F	Peritoneal mesothelioma	Chemotherapy	Discharge	Impossible	-	0	1	0	0	0	0	1	0	2	-	-	-	-	-	-	-	-	-
226	79	M	Large intestine	Adrenal cortical steroids	Discharge	Impossible	-	0	0	0	0	0	1	0	0	1	-	-	-	-	-	-	-	-	-
227	80	F	Lung	Chemotherapy	Discharge	Impossible	Impossible	0	1	0	0	0	0	0	0	1	0	0	0	0	0	0	1	0	1
228	80	F	Pancreas	Oral care	Death	Possible	Impossible	1	0	0	1	0	0	0	0	2	1	0	1	1	0	0	0	0	3
229	80	M	Bile duct, Gallbladder	Oral care	Discharge	Impossible	Impossible	0	0	1	1	0	0	2	0	4	0	0	1	1	0	0	2	0	4
230	80	M	Large intestine	Oral candidiasis	Death	Possible	Impossible	0	1	1	1	1	0	1	0	5	0	1	1	1	1	0	1	0	5
231	80	M	Urology	Oral care	Death	Impossible	-	0	1	0	0	0	0	1	0	2	-	-	-	-	-	-	-	-	-
232	80	M	Urology	Taste disorder	Discharge	Impossible	-	1	0	0	0	0	0	0	0	1	-	-	-	-	-	-	-	-	-
233	81	M	Lung	Oral care	Death	Impossible	Impossible	0	0	0	0	1	0	0	0	1	0	0	0	0	1	0	0	0	1
234	81	F	Lung	Oral care	Death	Possible	Impossible	0	0	0	0	0	0	1	0	1	0	0	0	0	0	0	1	0	1
235	81	M	Head and Neck	Adrenal cortical steroids	Discharge	Impossible	Impossible	0	0	0	0	0	0	0	0	0	0	1	0	0	0	0	0	0	1
236	81	F	Large intestine	Taste disorder	Death	Possible	Impossible	0	1	0	0	0	0	2	0	3	0	1	1	1	0	0	1	0	4
237	81	F	Large intestine	Adrenal cortical steroids	Discharge	Impossible	Impossible	0	0	0	0	0	0	0	0	0	0	1	1	1	0	0	1	0	4

238	81	M	Urology	Xerostomia	Death	Impossible	Impossible	1	1	1	2	0	0	1	0	6	1	1	1	1	0	0	1	0	5
239	81	F	Uterus, Ovaries	Oral care	Death	Impossible	Impossible	1	0	1	1	0	0	0	0	3	1	1	1	1	1	0	1	0	6
240	81	F	Urology	Pain	Discharge	Impossible	Impossible	0	0	0	1	0	0	0	1	2	0	1	1	1	0	2	1	0	6
241	81	M	Lung	Oral care	Death	Impossible	Impossible	0	0	0	1	0	0	0	0	1	1	1	1	2	0	0	2	0	7
242	81	M	Stomach	Oral care	Discharge	Impossible	Impossible	2	1	1	2	0	0	2	0	8	2	1	1	2	0	0	2	0	8
243	81	M	Stomach	Denture misfit	Death	Impossible	-	0	0	0	1	0	2	1	0	4	-	-	-	-	-	-	-	-	-
244	81	M	Bile duct, Gallbladder	Oral care	Death	Impossible	-	0	0	0	1	0	0	0	0	1	-	-	-	-	-	-	-	-	-
245	81	F	Lung	Chemotherapy	Discharge	Impossible	-	1	0	0	0	0	0	1	0	2	-	-	-	-	-	-	-	-	-
246	81	M	Stomach	Oral care	Discharge	Impossible	-	0	0	0	0	0	0	2	0	2	-	-	-	-	-	-	-	-	-
247	82	F	Lung	Osteoclastic inhibitor	Death	Impossible	Impossible	0	0	0	0	0	0	0	0	0	0	0	0	0	0	0	0	0	0
248	82	F	Pancreas	Oral care	Discharge	Impossible	Impossible	0	0	0	0	0	0	0	0	0	0	0	0	0	0	0	0	0	0
249	82	F	Pancreas	Chemotherapy	Discharge	Impossible	Impossible	0	1	0	0	0	0	0	0	1	0	1	0	0	0	0	0	0	1
250	82	F	Stomach	Adrenal cortical steroids	Discharge	Impossible	Impossible	0	1	0	0	0	0	0	0	1	0	1	0	0	0	0	0	0	1
251	82	F	Uterus, Ovaries	Oral care	Death	Impossible	Impossible	0	1	0	0	0	0	0	0	1	0	1	0	0	0	0	1	0	2
252	82	M	Stomach	Chemotherapy	Discharge	Impossible	Impossible	0	1	0	0	0	0	2	0	3	0	0	0	0	0	0	2	0	2
253	82	F	Large intestine	Xerostomia	Death	Impossible	Impossible	1	1	1	1	0	1	1	0	6	1	1	1	2	0	0	2	0	7
254	82	F	Stomach	Oral care	Discharge	Impossible	Impossible	1	1	1	2	0	0	1	0	6	1	1	2	2	0	0	2	0	8
255	82	M	Hepatocellular carcinoma	Xerostomia	Death	Impossible	-	2	1	0	1	0	0	1	0	5	-	-	-	-	-	-	-	-	-
256	82	M	Large intestine	Pain	Death	Impossible	-	1	1	2	1	0	0	0	0	5	-	-	-	-	-	-	-	-	-
257	82	F	Uterine sarcoma	Xerostomia	Discharge	Impossible	-	1	1	1	1	0	0	0	0	4	-	-	-	-	-	-	-	-	-

258	83	M	Unknown primary origin	Adrenal cortical steroids	Discharge	Impossible	Impossible	0	1	0	0	0	0	0	0	1	0	1	0	0	0	0	0	0	1
259	83	M	Urology	Pain	Death	Impossible	Impossible	0	0	0	0	0	0	0	0	0	0	1	0	1	0	0	1	0	3
260	83	F	Urology	Taste disorder	Death	Impossible	-	0	1	0	1	0	0	1	0	3	-	-	-	-	-	-	-	-	-
261	84	M	Stomach	Xerostomia	Discharge	Impossible	Impossible	0	0	0	0	0	0	0	0	0	0	0	0	0	0	0	0	0	0
262	84	M	Urology	Oral care	Discharge	Impossible	Impossible	0	0	0	0	0	0	1	0	1	0	0	0	0	0	0	1	0	1
263	84	M	Urology	Osteoclastic inhibitor	Discharge	Impossible	Impossible	0	0	0	0	0	0	1	1	2	0	0	0	0	0	0	1	0	1
264	84	M	Hepatocellular carcinoma	Oral care	Death	Impossible	Impossible	0	1	1	1	0	0	1	0	4	0	1	1	2	0	0	1	0	5
265	85	M	Urology	Tooth	Discharge	Possible	Impossible	0	1	0	0	0	0	0	0	1	0	0	0	0	0	0	0	0	0
266	85	M	Large intestine	Oral care	Discharge	Impossible	Impossible	0	0	0	0	0	0	0	0	0	0	0	0	0	0	0	0	0	0
267	85	F	Bile duct, Gallbladder	Xerostomia	Discharge	Impossible	Impossible	0	0	0	0	0	0	0	0	0	0	0	0	0	0	0	0	0	0
268	85	F	Stomach	Xerostomia	Discharge	Possible	Impossible	1	1	0	1	1	0	1	0	5	0	0	0	0	0	0	0	0	0
269	85	F	Peritoneal carcinoma	Xerostomia	Death	Impossible	Impossible	0	0	0	0	0	0	1	0	1	0	0	0	0	0	0	1	0	1
270	85	M	Large intestine	Oral candidiasis	Death	Impossible	Impossible	0	1	1	1	0	0	0	0	3	0	1	1	2	0	0	0	0	4
271	85	F	Large intestine	Xerostomia	Discharge	Impossible	Possible	1	0	0	0	0	0	0	0	1	1	2	0	1	0	0	1	0	5
272	85	F	Lung	Xerostomia	Discharge	Impossible	Impossible	1	1	1	1	1	0	2	0	7	0	0	1	1	1	1	1	0	5
273	85	F	Head and Neck	Pain	Discharge	Impossible	-	0	0	0	0	0	0	0	0	0	-	-	-	-	-	-	-	-	-
274	85	F	Breast	Oral care	Discharge	Impossible	-	0	0	1	1	0	0	0	0	2	-	-	-	-	-	-	-	-	-
275	86	M	Esophagus	Denture misfit	Discharge	Impossible	Impossible	0	0	0	0	0	2	0	0	2	0	0	0	0	0	0	0	0	0
276	86	M	Vulva Cancer S/O	Xerostomia	Discharge	Impossible	Impossible	0	0	0	0	0	0	0	0	0	0	0	0	0	0	0	0	0	0
277	86	M	Hepatocellular carcinoma	Oral care	Discharge	Impossible	Possible	0	0	0	0	1	0	1	0	2	0	0	0	0	1	0	1	0	2

278	86	M	Breast	Xerostomia	Death	Impossible	-	0	1	1	1	0	0	0	0	3	-	-	-	-	-	-	-	-	-
279	86	F	Lung	Adrenal cortical steroids	Discharge	Impossible	-	0	0	0	0	0	0	0	0	0	-	-	-	-	-	-	-	-	-
280	87	F	Large intestine	Oral care	Discharge	Impossible	Impossible	0	0	0	0	0	0	1	0	1	0	0	0	0	0	0	1	0	1
281	87	M	Lung	Adrenal cortical steroids	Death	Impossible	-	1	1	0	1	0	0	2	0	5	-	-	-	-	-	-	-	-	-
282	88	F	Lung	Oral care	Death	Impossible	Possible	0	0	1	1	0	0	1	0	3	0	0	0	0	0	1	1	0	2
283	88	F	Pancreas	Adrenal cortical steroids	Death	Impossible	-	0	0	0	0	0	0	2	0	2	-	-	-	-	-	-	-	-	-
284	89	M	Urology	Adrenal cortical steroids	Discharge	Possible	Possible	0	1	1	0	0	0	2	0	4	0	0	0	0	0	0	0	0	0
285	89	F	Malignant lymphoma	Oral care	Discharge	Possible	Impossible	0	2	1	2	0	0	1	0	6	0	0	0	0	0	0	0	0	0
286	89	M	Stomach	Xerostomia	Death	Impossible	Possible	1	1	0	0	0	0	0	0	2	1	1	0	0	0	0	0	0	2
287	89	M	Unknown primary origin	Oral care	Discharge	Impossible	Impossible	0	1	1	1	0	0	1	0	4	0	1	1	1	0	0	1	0	4
288	89	M	Urology	Oral care	Discharge	Possible	Impossible	0	1	1	1	0	0	1	0	4	0	1	1	1	0	0	1	0	4
289	89	M	Urology	Adrenal cortical steroids	Discharge	Impossible	Impossible	1	0	0	0	0	0	0	0	1	0	0	1	0	1	0	1	1	4
290	90	F	Head and Neck	Oral care	Death	Impossible	Impossible	1	0	1	0	0	0	2	0	4	1	0	1	1	0	0	2	0	5
291	90	M	Lung	Oral care	Death	Impossible	-	0	0	0	0	0	0	1	0	1	-	-	-	-	-	-	-	-	-
292	91	F	Pancreas	Oral care	Death	Possible	Impossible	0	0	1	0	0	0	0	0	1	0	0	1	0	0	0	0	0	1
293	91	M	Small intestine	Denture misfit	Discharge	Impossible	Impossible	0	1	0	0	0	0	1	0	2	0	1	0	0	0	0	1	0	2
294	91	F	Large intestine	Oral care	Discharge	Impossible	Impossible	0	1	0	0	1	0	1	0	3	0	1	0	0	1	0	1	0	3
295	91	F	Breast	Xerostomia	Death	Impossible	Impossible	0	1	2	1	0	0	2	0	6	0	1	2	1	0	0	2	0	6
296	92	F	Breast	Xerostomia	Death	Impossible	-	0	1	0	1	0	0	1	0	3	-	-	-	-	-	-	-	-	-
297	93	F	Urology	Xerostomia	Discharge	Impossible	-	1	1	0	1	0	0	0	1	4	-	-	-	-	-	-	-	-	-

298	93	M	Stomach	Oral care	Discharge	Impossible	-	0	1	1	1	0	0	0	0	3	-	-	-	-	-	-	-	-	-
299	95	M	Urology	Oral care	Discharge	Impossible	Impossible	0	0	0	0	0	0	0	0	0	0	0	0	0	0	0	0	0	0
300	100	F	Large intestine	Oral bleeding	Death	Impossible	Impossible	1	0	0	0	0	0	0	1	2	2	1	1	1	0	0	2	0	7
