# Supplementary material for: Longitudinal changes in oral conditions and oral candidiasis in palliative care inpatients: a longitudinal observational study
Source: Front Dent Med. 2026 Jul 2;7:1831411. doi: 10.3389/fdmed.2026.1831411 (PMC13372980; doi:10.3389/fdmed.2026.1831411)
Supplement: Supplementary file 3 [file Datasheet2.pdf]

Table S2 : Oral health status before and after oral care. Details of the 223 participants (Group A).

No.	(1) Age	(2) Sex	(3) Outcome	(4) Schedule			Oral candidiasis		OHAT (Before)									OHAT (After)								
				a Days	b Times	c Frequency (%)	Before	After	Lips	Tongue	Gums	Saliva	Natural teeth	Dentures	Oral cleanliness	Dental pain	Total score	Lips	Tongue	Gums	Saliva	Natural teeth	Dentures	Oral cleanliness	Dental pain	Total score
1	30	M	Death	24	8	33.3	Negative	Negative	1	0	0	0	1	0	1	0	3	0	0	0	2	0	2	0	0	4
2	44	F	Discharge	9	3	33.3	Negative	Negative	0	0	0	0	0	0	0	0	0	0	0	0	0	0	0	0	0	0
3	45	F	Death	11	3	27.3	Negative	Negative	1	1	0	0	0	0	0	0	2	1	1	1	1	0	0	0	0	4
4	46	F	Death	13	3	23.1	Negative	Negative	0	1	0	0	1	0	0	0	2	0	1	0	0	1	0	0	0	2
5	47	F	Discharge	21	4	19.0	Negative	Negative	1	1	1	1	0	0	0	0	4	0	0	0	0	0	0	0	0	0
6	47	F	Discharge	16	4	25.0	Negative	Negative	1	1	1	1	1	0	1	0	6	0	1	1	1	1	0	1	0	5
7	49	F	Discharge	24	7	29.2	Negative	Negative	0	0	0	0	0	0	0	0	0	0	0	0	0	0	0	0	0	0
8	49	M	Discharge	55	6	10.9	Negative	Negative	0	0	0	0	0	0	0	0	0	0	1	0	0	0	0	0	0	1
9	49	F	Discharge	8	2	25.0	Negative	Negative	0	0	0	0	0	0	0	0	0	1	1	0	1	0	0	0	0	3
10	49	F	Discharge	42	7	16.7	Negative	Negative	1	1	1	1	0	0	1	0	5	1	1	1	1	0	0	0	0	4
11	50	M	Discharge	8	2	25.0	Negative	Negative	0	0	0	0	0	0	0	0	0	0	0	0	0	0	0	0	0	0
12	50	F	Discharge	19	3	15.8	Negative	Negative	0	1	0	0	0	0	1	0	2	0	0	0	0	0	0	1	0	1
13	50	M	Death	12	3	25.0	Negative	Negative	0	1	0	0	1	0	1	0	3	0	1	0	1	1	0	0	0	3
14	50	M	Death	43	7	16.3	Negative	Negative	1	1	0	0	0	0	0	0	2	1	1	0	1	0	0	1	0	4
15	51	F	Discharge	10	2	20.0	Negative	Negative	1	0	0	0	0	0	0	0	1	1	0	0	0	0	0	0	0	1
16	53	M	Death	22	4	18.2	Negative	Negative	0	0	0	0	2	0	0	0	2	0	1	1	1	2	0	0	0	5
17	54	M	Discharge	60	8	13.3	Negative	Negative	0	0	0	0	0	0	1	0	1	0	0	0	0	2	0	1	0	3
18	55	M	Discharge	21	4	19.0	Negative	Negative	0	0	0	0	1	0	0	0	1	0	0	0	0	1	0	0	0	1
19	56	F	Discharge	57	9	15.8	Negative	Negative	0	0	0	0	0	0	0	0	0	0	0	0	0	0	0	0	0	0
20	56	M	Discharge	3	2	66.7	Negative	Negative	0	0	0	0	1	0	0	0	1	0	0	0	0	0	0	0	0	0
21	56	F	Discharge	9	3	33.3	Negative	Negative	0	1	0	0	0	0	1	0	2	0	1	0	0	0	0	0	0	1
22	56	M	Discharge	27	3	11.1	Negative	Negative	0	0	0	0	0	0	0	0	0	0	1	0	0	0	0	1	0	2
23	56	M	Death	19	5	26.3	Negative	Negative	0	1	0	0	0	0	0	0	1	0	1	0	0	0	0	2	0	3
24	56	M	Death	5	2	40.0	Negative	Negative	1	0	1	1	0	0	2	0	5	1	0	1	1	0	0	3	0	6
25	57	M	Discharge	5	3	60.0	Positive	Negative	0	1	0	1	0	0	1	0	3	0	1	0	1	0	0	1	0	3
26	58	M	Discharge	8	3	37.5	Negative	Negative	0	2	0	0	0	0	1	0	3	0	0	0	0	0	0	0	0	0
27	58	M	Discharge	3	2	66.7	Negative	Negative	0	1	1	1	0	0	1	0	4	0	1	0	0	0	0	0	0	1
28	59	M	Discharge	45	3	6.7	Negative	Negative	0	0	0	0	0	0	1	0	1	0	0	0	0	0	0	0	0	0
29	59	F	Discharge	19	6	31.6	Negative	Negative	0	0	0	1	0	0	0	0	1	0	1	0	1	0	0	0	0	2
30	59	M	Discharge	29	6	20.7	Negative	Negative	1	1	1	1	0	0	1	0	5	1	1	1	1	0	0	1	0	5
31	60	F	Discharge	34	4	11.8	Negative	Negative	0	1	0	0	0	0	0	0	1	0	0	0	0	0	0	0	0	0
32	60	M	Death	31	5	16.1	Negative	Negative	0	0	0	0	0	0	2	0	2	0	0	0	0	0	0	1	0	1
33	60	M	Death	40	9	22.5	Negative	Negative	0	0	0	0	0	0	0	0	0	0	0	0	1	0	0	0	0	1
34	60	M	Discharge	10	3	30.0	Negative	Negative	0	1	0	0	0	0	1	0	2	0	0	1	1	0	0	1	0	3
35	61	F	Death	8	2	25.0	Negative	Negative	1	0	1	1	0	0	0	0	3	1	0	1	1	0	0	0	0	0
36	61	M	Death	15	2	13.3	Negative	Negative	0	1	1	1	0	0	0	0	3	0	1	1	1	0	0	0	0	0
37	61	F	Death	5	2	40.0	Negative	Negative	0	1	0	0	0	0	0	1	2	0	1	0	0	0	0	0	1	2
38	61	M	Death	8	3	37.5	Negative	Negative	0	0	0	0	0	0	0	0	0	0	1	0	0	1	0	1	0	3
39	62	F	Death	2	2	100.0	Negative	Negative	0	0	0	0	1	1	0	1	3	0	0	0	0	0	0	0	0	0
40	62	F	Discharge	30	5	16.7	Negative	Negative	0	1	0	0	0	0	0	0	1	0	1	0	0	0	0	0	0	1
41	62	M	Discharge	26	8	30.8	Negative	Negative	0	0	0	0	0	0	0	0	0	0	1	1	1	0	0	0	0	3
42	63	M	Discharge	59	3	5.1	Negative	Negative	0	0	0	0	0	0	0	0	0	0	0	0	0	0	0	0	0	0
43	63	M	Discharge	19	5	26.3	Negative	Negative	0	1	0	0	0	0	2	0	3	0	0	0	0	0	0	1	0	1
44	63	M	Death	9	4	44.4	Negative	Negative	0	1	1	1	2	0	1	0	6	0	1	1	1	2	0	1	0	6
45	64	M	Discharge	59	9	15.3	Negative	Negative	0	0	0	1	0	0	0	0	1	0	0	0	0	0	0	0	0	0
46	64	M	Discharge	53	5	9.4	Negative	Negative	0	0	0	0	1	0	0	0	1	0	0	0	0	1	0	0	0	1
47	64	M	Discharge	41	6	14.6	Negative	Negative	0	1	0	1	0	0	1	0	3	0	1	0	0	0	0	0	0	1
48	64	M	Death	29	7	24.1	Negative	Negative	0	0	0	1	0	0	0	0	1	0	0	0	2	0	0	0	0	2
49	64	M	Death	15	3	20.0	Negative	Negative	1	1	1	1	0	0	0	0	4	0	1	1	1	0	0	0	0	3
50	64	M	Death	17	5	29.4	Negative	Positive	0	0	0	0	0	2	2	0	4	0	1	0	1	1	0	1	0	4
51	64	M	Discharge	28	7	25.0	Negative	Negative	0	1	2	1	1	2	2	0	9	0	1	2	1	1	2	2	0	9
52	65	F	Death	8	4	50.0	Negative	Negative	0	1	0	0	0	0	1	0	2	0	1	0	0	0	0	0	0	1
53	65	F	Death	2	2	100.0	Negative	Negative	0	0	0	0	0	0	0	0	0	2	1	0	0	0	0	0	0	3
54	65	M	Discharge	9	4	44.4	Negative	Positive	0	1	1	1	0	0	0	0	3	0	1	1	1	0	0	0	0	3
55	65	F	Discharge	7	3	42.9	Negative	Negative	1	0	0	0	0	0	0	0	1	1	0	1	1	0	0	1	0	4
56	65	M	Death	11	6	54.5	Negative	Negative	1	0	1	0	2	0	2	0	6	0	1	2	2	2	0	1	0	8
57	66	F	Discharge	8	2	25.0	Negative	Positive	0	0	0	0	0	0	0	0	0	0	0	0	0	0	0	0	0	0
58	66	M	Discharge	57	10	17.5	Negative	Negative	0	2	2	0	0	0	1	0	5	0	0	0	0	0	0	0	0	0
59	66	M	Death	10	3	30.0	Negative	Negative	0	0	0	0	1	0	0	0	1	0	0	0	0	0	0	1	0	1
60	66	F	Discharge	6	2	33.3	Negative	Negative	0	0	0	0	1	0	0	0	1	0	0	0	0	1	0	0	0	1
61	66	M	Discharge	4	3	75.0	Negative	Negative	0	1	0	1	0	0	1	0	3	0	1	0	0	0	0	0	0	1
62	66	M	Death	11	3	27.3	Negative	Negative	0	1	1	1	0	0	0	0	3	1	1	1	1	0	0	0	0	4
63	66	M	Discharge	2	2	100.0	Negative	Negative	0	1	0	0	0	0	1	0	2	0	1	1	1	0	0	1	0	4
64	66	M	Discharge	11	7	63.6	Negative	Negative	1	1	1	2	0	0	1	0	6	1	1	1	2	0	0	1	0	6
65	67	F	Discharge	6	2	33.3	Negative	Negative	1	0	0	0	0	0	0	0	1	0	0	0	0	0	0	0	0	0
66	67	F	Discharge	7	2	28.6	Negative	Negative	0	0	0	0	0	0	0	0	0	0	0	0	0	0	0	0	0	0
67	67	M	Death	31	8	25.8	Positive	Negative	0	1	1	0	1	0	1	0	4	0	0	1	0	0	0	0	0	1
68	67	F	Discharge	59	4	6.8	Negative	Positive	0	0	0	1	0	0	2	0	3	0	0	0	1	0	0	0	0	1
69	67	M	Death	6	3	50.0	Negative	Negative	0	0	0	0	0	0	0	1	1	0	1	1	1	0	0	0	0	3
70	67	M	Death	18	8	44.4	Negative	Negative	1	1	0	0	0	0	0	0	2	0	1	1	1	0	0	1	0	4
71	67	M	Death	8	2	25.0	Positive	Negative	0	0	1	1	0	0	2	0	4	0	0	1	1	0	0	2	0	4
72	67	M	Discharge	57	7	12.3	Positive	Negative	0	1	1	1	2	0	2	0	7	0	1	0	0	2	0	1	0	4
73	67	F	Discharge	46	6	13.0	Negative	Negative	0	0	0	0	0	0	0	0	0	1	1	1	1	0	0	0	0	4
74	67	M	Discharge	2	2	100.0	Negative	Negative	0	1	1	1	1	0	1	0	5	0	1	1	1	1	0	1	0	5
75	67	M	Discharge	36	8	22.2	Negative	Negative	1	0	2	1	2	0	2	2	10	1	0	2	1	2	0	1	0	7
76	68	F	Discharge	59	6	10.2	Negative	Negative	0	0	0	0	0	0	0	0	0	0	0	0	0	0	0	0	0	0
77	68	M	Discharge	16	3	18.8	Negative	Negative	1	0	0	2	0	0	0	2	5	0	1	0	0	0	0	1	0	2
78	68	F	Discharge	9	3	33.3	Negative	Negative	0	1	0	0	0	0	2	0	3	0	1	0	0	0	0	1	0	2
79	68	F	Death	46	4	8.7	Negative	Negative	0	0	0	0	1	0	0	0	1	0	0	1	1	1	0	0	0	3

80	68	M	Discharge	7	3	42.9	Negative	Negative	0	1	0	0	1	0	1	0	3	0	1	0	0	1	0	1	0	3
81	68	M	Discharge	13	6	46.2	Positive	Negative	0	2	2	1	0	0	2	1	8	0	0	1	1	0	0	1	0	3
82	68	M	Death	40	8	20.0	Negative	Negative	0	1	0	0	1	0	1	0	3	0	1	1	1	1	0	0	0	4
83	69	M	Death	6	2	33.3	Negative	Negative	0	0	0	0	1	0	0	0	1	0	0	0	0	0	0	2	0	2
84	69	F	Discharge	8	2	25.0	Negative	Negative	0	0	0	0	0	2	0	0	2	0	0	0	0	0	0	2	0	2
85	69	F	Discharge	31	8	25.8	Negative	Negative	1	0	0	0	1	0	2	0	4	1	0	0	0	1	0	1	0	3
86	69	F	Discharge	4	3	75.0	Negative	Negative	2	1	2	0	0	0	1	1	7	1	1	1	1	0	0	1	0	5
87	70	M	Discharge	15	3	20.0	Negative	Negative	0	0	0	0	0	0	0	0	0	0	0	0	0	0	0	0	0	0
88	70	M	Discharge	22	3	13.6	Positive	Negative	0	0	0	0	0	0	1	0	1	0	0	0	0	0	0	0	0	0
89	70	M	Death	9	2	22.2	Negative	Negative	1	0	0	0	0	0	0	0	1	1	0	0	0	0	0	0	0	1
90	70	F	Death	15	5	33.3	Negative	Negative	0	0	0	0	0	0	0	0	0	0	0	0	1	0	0	0	0	1
91	70	F	Discharge	18	2	11.1	Negative	Negative	0	1	0	0	0	0	1	0	2	0	0	0	0	0	0	1	0	1
92	70	M	Discharge	21	5	23.8	Negative	Negative	0	1	0	0	0	0	0	0	1	0	1	0	0	0	0	0	0	1
93	70	F	Discharge	10	5	50.0	Negative	Negative	0	0	0	0	0	0	1	0	1	0	0	0	0	1	0	1	0	2
94	70	F	Discharge	8	3	37.5	Negative	Negative	1	1	0	2	0	0	1	0	5	0	0	1	0	0	1	0	0	2
95	70	M	Discharge	23	6	26.1	Negative	Negative	1	0	0	0	0	0	1	0	2	0	0	1	1	0	0	1	0	3
96	70	M	Discharge	8	2	25.0	Negative	Negative	0	1	0	0	2	0	1	0	4	0	1	0	0	2	1	1	0	5
97	70	F	Death	2	2	100.0	Negative	Negative	1	1	1	1	1	0	1	0	6	1	1	1	1	1	0	1	0	6
98	71	M	Death	29	2	6.9	Negative	Negative	0	0	0	1	0	0	1	0	2	0	0	0	0	0	0	0	0	0
99	71	M	Death	8	3	37.5	Negative	Negative	1	1	1	1	0	0	0	0	4	1	2	1	1	0	0	0	0	0
100	71	F	Discharge	4	3	75.0	Negative	Negative	0	0	2	0	0	0	1	0	3	0	0	0	0	0	0	0	0	0
101	71	M	Death	18	5	27.8	Negative	Negative	0	0	0	0	0	0	1	0	1	0	0	1	0	0	0	1	0	2
102	71	M	Discharge	14	4	28.6	Negative	Negative	0	0	0	0	0	2	0	0	2	0	0	0	0	0	2	0	0	2
103	71	M	Death	8	3	37.5	Negative	Negative	0	1	1	1	0	0	1	0	4	0	1	1	1	0	0	0	0	3
104	71	M	Discharge	29	6	20.7	Positive	Positive	1	1	1	1	0	0	2	0	6	0	0	1	1	0	0	1	0	3
105	71	M	Death	20	7	35.0	Positive	Negative	2	1	2	1	0	0	1	0	7	1	2	2	0	0	0	1	0	6
106	72	M	Death	4	2	50.0	Negative	Negative	0	0	1	1	0	0	1	0	3	0	0	0	0	0	0	0	0	0
107	72	M	Discharge	28	5	17.9	Negative	Negative	0	1	0	0	0	1	1	0	3	0	1	0	0	0	0	0	0	1
108	72	M	Death	3	2	66.7	Negative	Negative	0	1	0	0	0	0	1	0	2	0	0	1	1	0	0	0	0	2
109	72	M	Discharge	2	2	100.0	Negative	Negative	0	1	0	0	0	0	1	0	2	0	1	0	0	0	0	1	0	2
110	72	F	Death	2	2	100.0	Negative	Negative	0	1	1	1	0	0	0	0	3	0	1	1	1	0	0	0	0	3
111	72	F	Discharge	3	2	66.7	Negative	Positive	1	0	1	1	0	0	0	1	4	1	0	1	0	0	1	0	0	3
112	72	F	Death	11	4	36.4	Negative	Negative	0	0	0	0	1	2	1	0	4	0	0	0	0	1	2	1	0	4
113	72	F	Discharge	5	2	40.0	Negative	Negative	0	1	1	1	0	0	1	0	4	0	1	1	1	0	0	1	0	4
114	72	M	Discharge	16	5	31.3	Negative	Negative	0	0	0	0	0	0	0	0	0	1	1	1	1	0	0	1	0	5
115	72	M	Discharge	7	2	28.6	Negative	Positive	1	1	1	1	0	0	1	0	5	0	1	1	1	0	0	2	0	5
116	72	M	Death	15	5	33.3	Negative	Negative	1	0	2	0	0	0	0	0	3	1	0	1	1	0	2	0	2	7
117	73	F	Discharge	9	4	44.4	Positive	Negative	0	0	0	0	0	0	0	0	0	0	0	0	0	0	0	0	0	0
118	73	F	Discharge	12	3	25.0	Negative	Negative	0	0	0	0	0	0	0	0	0	0	0	0	0	0	0	0	0	0
119	73	M	Discharge	7	4	57.1	Positive	Positive	0	0	0	0	0	0	0	0	0	0	0	0	0	0	0	0	0	0
120	73	F	Discharge	18	2	11.1	Negative	Negative	0	0	0	0	0	0	1	0	1	0	0	0	0	0	0	0	0	0
121	73	M	Death	23	4	17.4	Positive	Positive	0	0	0	0	1	0	2	0	3	0	0	0	1	0	0	1	0	2
122	73	M	Discharge	6	3	50.0	Negative	Negative	0	0	1	1	0	0	0	0	2	0	0	1	1	0	0	0	0	2
123	73	M	Discharge	8	2	25.0	Negative	Negative	0	0	0	0	1	0	1	0	2	0	0	0	0	1	0	1	0	2
124	73	F	Discharge	8	3	37.5	Negative	Negative	0	0	0	0	2	0	1	0	3	0	0	0	0	2	0	1	0	3
125	73	F	Death	59	10	16.9	Positive	Negative	0	0	0	0	1	0	0	0	1	0	1	0	0	1	0	2	0	4
126	73	F	Death	2	2	100.0	Negative	Negative	0	0	1	1	0	0	1	0	3	0	1	1	1	0	0	1	0	4
127	73	M	Discharge	7	2	28.6	Negative	Negative	0	0	0	0	0	0	0	0	0	0	1	1	1	0	0	1	0	4
128	73	M	Death	7	2	28.6	Positive	Negative	1	1	0	0	0	0	0	0	2	1	1	1	1	0	0	1	0	5
129	73	M	Death	5	4	80.0	Positive	Negative	1	1	1	1	0	0	2	0	6	0	1	1	2	0	0	1	0	5
130	74	F	Discharge	3	2	66.7	Negative	Negative	0	0	0	0	0	0	0	0	0	0	0	0	0	0	0	0	0	0
131	74	F	Discharge	11	5	45.5	Positive	Negative	0	0	0	0	0	0	0	0	0	0	0	0	0	0	0	0	0	0
132	74	F	Discharge	13	3	23.1	Positive	Negative	1	0	0	0	0	0	0	0	1	1	0	0	0	0	0	0	0	1
133	74	F	Death	27	4	14.8	Negative	Negative	0	1	0	0	1	0	2	0	4	0	1	0	1	0	1	0	0	3
134	74	M	Death	15	2	13.3	Negative	Negative	0	1	1	1	0	0	2	0	5	0	1	1	1	0	0	1	0	4
135	74	F	Death	3	3	100.0	Negative	Negative	1	1	1	1	1	0	1	1	7	0	1	0	1	1	0	1	0	4
136	74	F	Discharge	2	2	100.0	Negative	Negative	1	1	1	1	0	1	1	0	6	0	1	1	2	0	1	1	0	6
137	75	M	Discharge	26	6	23.1	Negative	Negative	0	1	0	0	0	0	1	0	2	0	0	0	0	0	0	0	0	0
138	75	F	Discharge	13	4	30.8	Negative	Negative	0	0	0	0	0	0	0	0	0	0	0	0	0	0	0	0	0	0
139	75	F	Death	8	2	25.0	Negative	Negative	0	1	0	0	0	0	0	0	1	0	1	0	0	0	0	0	0	1
140	75	M	Death	11	3	27.3	Negative	Negative	0	1	1	0	0	0	0	0	2	0	1	1	0	0	0	0	0	2
141	75	M	Discharge	7	2	28.6	Negative	Negative	0	1	0	0	0	0	1	0	2	0	1	0	0	0	0	1	0	2
142	75	F	Death	4	2	50.0	Negative	Negative	0	1	1	1	0	0	0	1	4	0	1	1	1	0	0	0	0	3
143	75	F	Death	10	2	20.0	Negative	Negative	0	0	0	0	0	0	0	0	0	0	1	1	1	0	0	0	0	3
144	75	M	Death	3	2	66.7	Negative	Negative	1	1	0	1	0	0	1	0	4	1	1	0	1	0	0	1	0	4
145	75	M	Discharge	4	4	100.0	Positive	Negative	0	0	0	0	0	0	0	0	0	0	1	1	1	0	0	1	0	4
146	76	F	Discharge	12	3	25.0	Negative	Negative	0	1	0	0	0	0	0	0	1	0	0	0	0	0	0	0	0	0
147	76	M	Discharge	20	7	35.0	Negative	Negative	0	1	0	1	0	0	0	0	2	0	0	0	0	0	0	0	0	0
148	76	F	Discharge	15	4	26.7	Negative	Negative	0	1	0	0	2	1	2	0	6	0	0	0	0	0	0	0	0	0
149	76	M	Death	17	6	35.3	Negative	Negative	0	0	0	0	0	0	1	0	1	0	0	0	0	0	0	1	0	1
150	76	F	Discharge	14	4	28.6	Negative	Negative	0	1	0	0	1	0	1	0	3	0	0	0	0	1	0	1	0	2
151	76	M	Death	5	2	40.0	Negative	Negative	1	0	1	1	0	0	0	0	3	1	0	1	1	0	0	0	0	3
152	77	F	Discharge	29	5	17.2	Negative	Negative	0	0	0	0	0	0	0	0	0	0	0	0	0	0	0	0	0	0
153	77	M	Discharge	14	14	100.0	Negative	Negative	0	0	0	0	0	0	1	0	1	0	0	0	0	0	0	0	0	0
154	77	M	Discharge	22	4	18.2	Negative	Negative	0	1	0	0	0	0	1	0	2	0	1	0	0	0	0	1	0	2
155	77	M	Discharge	4	2	50.0	Positive	Negative	0	0	0	0	0	2	0	0	2	0	0	0	0	0	2	0	0	2
156	77	M	Death	24	7	29.2	Negative	Negative	0	0	1	1	1	0	0	0	3	0	0	0	1	1	0	2	0	4
157	77	M	Discharge	6	2	33.3	Negative	Negative	0	1	1	1	0	0	1	0	4	0	1	1	1	0	0	1	0	4
158	77	M	Discharge	14	4	28.6	Negative	Negative	0	1	1	1	0	0	1	0	4	0	1	1	1	0	0	1	0	4
159	77	M	Discharge	14	4	28.6	Negative	Negative	0	0	1	0	0	0	2	0	3	1	1	1	0	0	0	1	0	4
160	77	M	Death	16	5	31.3	Negative	Negative	0	1	0	0	2	0	2	0	5	1	1	0	0	2	0	2	2	8
161	78	F	Discharge	2	2	100.0	Negative	Negative	2	0	0	0	0	0	0	0	2	0	0	0	0	0	0	0	0	0
162	78	F	Death	3	2	66.7	Negative	Negative	1	0	1	1	0	0	1	0	4	0	0	0	1	0	0	0	0	1
163	78	M	Death	28	4	14.3	Negative	Negative	0	0	0	0	0	0	0	0	0	0	0	0	0	0	0	1	0	1
164	78	M	Discharge	5	2	40.0	Negative	Positive	0	0	0	0	0	0	1	0	1	0	0	0	0	0	0	1	0	1
165	78	M	Death	3	2	66.7	Positive	Negative	1	1	1	1	0	0	1	0	5	1	1	1	1	0	0	1	0	5
166	78	M	Discharge	3	2	66.7	Negative	Negative	0	0	1	1	2	2	1	0	7	0	0	1	1	2	2	0	0	6

167	79	M	Discharge	37	4	10.8	Negative	Negative	0	0	0	0	0	0	0	0	0	0	0	0	0	0	0	0	0	0
168	79	F	Discharge	26	4	15.4	Negative	Negative	0	0	0	0	0	0	0	0	0	0	0	0	0	0	0	0	0	0
169	79	M	Discharge	20	3	15.0	Negative	Positive	0	0	0	0	0	0	1	0	1	0	1	1	1	0	0	1	0	4
170	80	F	Discharge	13	4	30.8	Negative	Negative	0	1	0	0	0	0	0	0	1	0	0	0	0	0	0	1	0	1
171	80	F	Death	8	3	37.5	Positive	Negative	1	0	0	1	0	0	0	0	2	1	0	1	1	0	0	0	0	3
172	80	M	Discharge	10	2	20.0	Negative	Negative	0	0	1	1	0	0	2	0	4	0	0	1	1	0	0	2	0	4
173	80	M	Death	2	2	100.0	Positive	Negative	0	1	1	1	1	0	1	0	5	0	1	1	1	1	0	1	0	5
174	81	M	Death	5	2	40.0	Negative	Negative	0	0	0	0	1	0	0	0	1	0	0	0	0	1	0	0	0	1
175	81	F	Death	8	2	25.0	Positive	Negative	0	0	0	0	0	0	1	0	1	0	0	0	0	0	0	1	0	1
176	81	M	Discharge	20	4	20.0	Negative	Negative	0	0	0	0	0	0	0	0	0	0	1	0	0	0	0	0	0	1
177	81	F	Death	10	3	30.0	Positive	Negative	0	1	0	0	0	0	2	0	3	0	1	1	1	0	0	1	0	4
178	81	F	Discharge	4	2	50.0	Negative	Negative	0	0	0	0	0	0	0	0	0	0	1	1	1	0	0	1	0	4
179	81	M	Death	15	5	33.3	Negative	Negative	1	1	1	2	0	0	1	0	6	1	1	1	1	0	0	1	0	5
180	81	F	Death	52	8	15.4	Negative	Negative	1	0	1	1	0	0	0	0	3	1	1	1	1	1	0	1	0	6
181	81	F	Discharge	12	5	41.7	Negative	Negative	0	0	0	1	0	0	0	1	2	0	1	1	1	0	2	1	0	6
182	81	M	Death	11	5	45.5	Negative	Negative	0	0	0	1	0	0	0	0	1	1	1	1	2	0	0	2	0	7
183	81	M	Discharge	10	2	20.0	Negative	Negative	2	1	1	2	0	0	2	0	8	2	1	1	2	0	0	2	0	8
184	82	F	Death	12	3	25.0	Negative	Negative	0	0	0	0	0	0	0	0	0	0	0	0	0	0	0	0	0	0
185	82	F	Discharge	8	2	25.0	Negative	Negative	0	0	0	0	0	0	0	0	0	0	0	0	0	0	0	0	0	0
186	82	F	Discharge	59	6	10.2	Negative	Negative	0	1	0	0	0	0	0	0	1	0	1	0	0	0	0	0	0	1
187	82	F	Discharge	5	2	40.0	Negative	Negative	0	1	0	0	0	0	0	0	1	0	1	0	0	0	0	0	0	1
188	82	F	Death	15	3	20.0	Negative	Negative	0	1	0	0	0	0	0	0	1	0	1	0	0	0	0	1	0	2
189	82	M	Discharge	31	8	25.8	Negative	Negative	0	1	0	0	0	0	2	0	3	0	0	0	0	0	0	2	0	2
190	82	F	Death	5	3	60.0	Negative	Negative	1	1	1	1	0	1	1	0	6	1	1	1	2	0	0	2	0	7
191	82	F	Discharge	7	4	57.1	Negative	Negative	1	1	1	2	0	0	1	0	6	1	1	2	2	0	0	2	0	8
192	83	M	Discharge	8	2	25.0	Negative	Negative	0	1	0	0	0	0	0	0	1	0	1	0	0	0	0	0	0	1
193	83	M	Death	21	7	33.3	Negative	Negative	0	0	0	0	0	0	0	0	0	0	1	0	1	0	0	1	0	3
194	84	M	Discharge	36	8	22.2	Negative	Negative	0	0	0	0	0	0	0	0	0	0	0	0	0	0	0	0	0	0
195	84	M	Discharge	11	3	27.3	Negative	Negative	0	0	0	0	0	0	1	0	1	0	0	0	0	0	0	1	0	1
196	84	M	Discharge	27	8	29.6	Negative	Negative	0	0	0	0	0	0	1	1	2	0	0	0	0	0	0	1	0	1
197	84	M	Death	4	2	50.0	Negative	Negative	0	1	1	1	0	0	1	0	4	0	1	1	2	0	0	1	0	5
198	85	M	Discharge	8	3	37.5	Positive	Negative	0	1	0	0	0	0	0	0	1	0	0	0	0	0	0	0	0	0
199	85	M	Discharge	14	3	21.4	Negative	Negative	0	0	0	0	0	0	0	0	0	0	0	0	0	0	0	0	0	0
200	85	F	Discharge	7	3	42.9	Negative	Negative	0	0	0	0	0	0	0	0	0	0	0	0	0	0	0	0	0	0
201	85	F	Discharge	8	3	37.5	Positive	Negative	1	1	0	1	1	0	1	0	5	0	0	0	0	0	0	0	0	0
202	85	F	Death	13	5	38.5	Negative	Negative	0	0	0	0	0	0	1	0	1	0	0	0	0	0	0	1	0	1
203	85	M	Death	13	4	30.8	Negative	Negative	0	1	1	1	0	0	0	0	3	0	1	1	2	0	0	0	0	4
204	85	F	Discharge	14	4	28.6	Negative	Positive	1	0	0	0	0	0	0	0	1	1	2	0	1	0	0	1	0	5
205	85	F	Discharge	5	3	60.0	Negative	Negative	1	1	1	1	1	0	2	0	7	0	0	1	1	1	1	1	0	5
206	86	M	Discharge	7	3	42.9	Negative	Negative	0	0	0	0	0	2	0	0	2	0	0	0	0	0	0	0	0	0
207	86	M	Discharge	14	2	14.3	Negative	Negative	0	0	0	0	0	0	0	0	0	0	0	0	0	0	0	0	0	0
208	86	M	Discharge	4	2	50.0	Negative	Positive	0	0	0	0	1	0	1	0	2	0	0	0	0	1	0	1	0	2
209	87	F	Discharge	15	3	20.0	Negative	Negative	0	0	0	0	0	0	1	0	1	0	0	0	0	0	0	1	0	1
210	88	F	Death	22	6	27.3	Negative	Positive	0	0	1	1	0	0	1	0	3	0	0	0	0	0	1	1	0	2
211	89	M	Discharge	3	2	66.7	Positive	Positive	0	1	1	0	0	0	2	0	4	0	0	0	0	0	0	0	0	0
212	89	F	Discharge	28	5	17.9	Positive	Negative	0	2	1	2	0	0	1	0	6	0	0	0	0	0	0	0	0	0
213	89	M	Death	7	3	42.9	Negative	Positive	1	1	0	0	0	0	0	0	2	1	1	0	0	0	0	0	0	2
214	89	M	Discharge	28	4	14.3	Negative	Negative	0	1	1	1	0	0	1	0	4	0	1	1	1	0	0	1	0	4
215	89	M	Discharge	14	6	42.9	Positive	Negative	0	1	1	1	0	0	1	0	4	0	1	1	1	0	0	1	0	4
216	89	M	Discharge	2	2	100.0	Negative	Negative	1	0	0	0	0	0	0	0	1	0	0	1	0	1	0	1	1	4
217	90	F	Death	3	2	66.7	Negative	Negative	1	0	1	0	0	0	2	0	4	1	0	1	1	0	0	2	0	5
218	91	F	Death	27	5	18.5	Positive	Negative	0	0	1	0	0	0	0	0	1	0	0	1	0	0	0	0	0	1
219	91	M	Discharge	6	2	33.3	Negative	Negative	0	1	0	0	0	0	1	0	2	0	1	0	0	0	0	1	0	2
220	91	F	Discharge	2	2	100.0	Negative	Negative	0	1	0	0	1	0	1	0	3	0	1	0	0	1	0	1	0	3
221	91	F	Death	3	2	66.7	Negative	Negative	0	1	2	1	0	0	2	0	6	0	1	2	1	0	0	2	0	6
222	95	M	Discharge	7	3	42.9	Negative	Negative	0	0	0	0	0	0	0	0	0	0	0	0	0	0	0	0	0	0
223	100	F	Death	33	16	48.5	Negative	Negative	1	0	0	0	0	0	0	1	2	2	1	1	1	0	0	2	0	7
