# Supplementary material for: Longitudinal changes in oral conditions and oral candidiasis in palliative care inpatients: a longitudinal observational study
Source: Front Dent Med. 2026 Jul 2;7:1831411. doi: 10.3389/fdmed.2026.1831411 (PMC13372980; doi:10.3389/fdmed.2026.1831411)
Supplement: Supplementary file 4 [file Datasheet3.pdf]

Table S3 : Details of deceased participants' oral health (Group ND, n= 85; Group NX, n=30).

GroupND																						
No.	(1) Age	(2)Sex	(3) Oral candidiasis		(4)OHAT (Before)									(4) OHAT (After)								
			Before	After	Lips	Tongue	Gums	Saliva	Natural teeth	Dentures	Oral cleanliness	Dental pain	Total score	Lips	Tongue	Gums	Saliva	Natural teeth	Dentures	Oral cleanliness	Dental pain	Total score
1	30	M	Negative	Negative	1	0	0	0	1	0	1	0	3	0	0	0	2	0	2	0	0	4
2	45	F	Negative	Negative	1	1	0	0	0	0	0	0	2	1	1	1	1	0	0	0	0	4
3	46	F	Negative	Negative	0	1	0	0	1	0	0	0	2	0	1	0	0	1	0	0	0	2
4	50	M	Negative	Negative	1	1	0	0	0	0	0	0	2	1	1	0	1	0	0	1	0	4
5	50	M	Negative	Negative	0	1	0	0	1	0	1	0	3	0	1	0	1	1	0	0	0	3
6	53	M	Negative	Negative	0	0	0	0	2	0	0	0	2	0	1	1	1	2	0	0	0	5
7	56	M	Negative	Negative	0	1	0	0	0	0	0	0	1	0	1	0	0	0	0	2	0	3
8	56	M	Negative	Negative	1	0	1	1	0	0	2	0	5	1	0	1	1	0	0	3	0	6
9	60	M	Negative	Negative	0	0	0	0	0	0	2	0	2	0	0	0	0	0	0	1	0	1
10	60	M	Negative	Negative	0	0	0	0	0	0	0	0	0	0	0	0	1	0	0	0	0	1
11	61	F	Negative	Negative	0	1	0	0	0	0	0	1	2	0	1	0	0	0	0	0	1	2
12	61	M	Negative	Negative	0	0	0	0	0	0	0	0	0	0	1	0	0	1	0	1	0	3
13	61	M	Negative	Negative	0	1	1	1	0	0	0	0	3	0	1	1	1	0	0	0	0	0
14	61	F	Negative	Negative	1	0	1	1	0	0	0	0	3	1	0	1	1	0	0	0	0	0
15	62	F	Negative	Negative	0	0	0	0	1	1	0	1	3	0	0	0	0	0	0	0	0	0
16	63	M	Negative	Negative	0	1	1	1	2	0	1	0	6	0	1	1	1	2	0	1	0	6
17	64	M	Negative	Positive	0	0	0	0	0	2	2	0	4	0	1	0	1	1	0	1	0	4
18	64	M	Negative	Negative	1	1	1	1	0	0	0	0	4	0	1	1	1	0	0	0	0	3
19	64	M	Negative	Negative	0	0	0	1	0	0	0	0	1	0	0	0	2	0	0	0	0	2
20	65	M	Negative	Negative	1	0	1	0	2	0	2	0	6	0	1	2	2	2	0	1	0	8
21	65	F	Negative	Negative	0	1	0	0	0	0	1	0	2	0	1	0	0	0	0	0	0	1
22	65	F	Negative	Negative	0	0	0	0	0	0	0	0	0	2	1	0	0	0	0	0	0	3
23	66	M	Negative	Negative	0	1	1	1	0	0	0	0	3	1	1	1	1	0	0	0	0	4
24	66	M	Negative	Negative	0	0	0	0	1	0	0	0	1	0	0	0	0	0	0	1	0	1
25	67	M	Negative	Negative	1	1	0	0	0	0	0	0	2	0	1	1	1	0	0	1	0	4
26	67	M	Positive	Negative	0	0	1	1	0	0	2	0	4	0	0	1	1	0	0	2	0	4
27	67	M	Negative	Negative	0	0	0	0	0	0	0	1	1	0	1	1	1	0	0	0	0	3
28	67	M	Positive	Negative	0	1	1	0	1	0	1	0	4	0	0	1	0	0	0	0	0	1
29	68	F	Negative	Negative	0	0	0	0	1	0	0	0	1	0	0	1	1	1	0	0	0	3
30	68	M	Negative	Negative	0	1	0	0	1	0	1	0	3	0	1	1	1	1	0	0	0	4
31	69	M	Negative	Negative	0	0	0	0	1	0	0	0	1	0	0	0	0	0	0	2	0	2
32	70	M	Negative	Negative	1	0	0	0	0	0	0	0	1	1	0	0	0	0	0	0	0	1
33	70	F	Negative	Negative	1	1	1	1	1	0	1	0	6	1	1	1	1	1	0	1	0	6
34	70	F	Negative	Negative	0	0	0	0	0	0	0	0	0	0	0	0	1	0	0	0	0	1
35	71	M	Negative	Negative	0	0	0	1	0	0	1	0	2	0	0	0	0	0	0	0	0	0
36	71	M	Negative	Negative	0	0	0	0	0	0	1	0	1	0	0	1	0	0	0	1	0	2
37	71	M	Negative	Negative	0	1	1	1	0	0	1	0	4	0	1	1	1	0	0	0	0	3
38	71	M	Positive	Negative	2	1	2	1	0	0	1	0	7	1	2	2	0	0	0	1	0	6
39	71	M	Negative	Negative	1	1	1	1	0	0	0	0	4	1	2	1	1	0	0	0	0	0
40	72	F	Negative	Negative	0	0	0	0	1	2	1	0	4	0	0	0	0	1	2	1	0	4
41	72	M	Negative	Negative	0	1	0	0	0	0	1	0	2	0	0	1	1	0	0	0	0	2
42	72	M	Negative	Negative	0	0	1	1	0	0	1	0	3	0	0	0	0	0	0	0	0	0
43	72	F	Negative	Negative	0	1	1	1	0	0	0	0	3	0	1	1	1	0	0	0	0	3
44	72	M	Negative	Negative	1	0	2	0	0	0	0	0	3	1	0	1	1	0	2	0	2	7
45	73	M	Positive	Negative	1	1	0	0	0	0	0	0	2	1	1	1	1	0	0	1	0	5
46	73	F	Positive	Negative	0	0	0	0	1	0	0	0	1	0	1	0	0	1	0	2	0	4
47	73	F	Negative	Negative	0	0	1	1	0	0	1	0	3	0	1	1	1	0	0	1	0	4
48	73	M	Positive	Negative	1	1	1	1	0	0	2	0	6	0	1	1	2	0	0	1	0	5
49	73	M	Positive	Positive	0	0	0	0	1	0	2	0	3	0	0	0	1	0	0	1	0	2
50	74	M	Negative	Negative	0	1	1	1	0	0	2	0	5	0	1	1	1	0	0	1	0	4
51	74	F	Negative	Negative	1	1	1	1	1	0	1	1	7	0	1	0	1	1	0	1	0	4
52	74	F	Negative	Negative	0	1	0	0	1	0	2	0	4	0	1	0	1	0	1	0	0	3

53	75	M	Negative	Negative	0	1	1	0	0	0	0	0	2	0	1	1	0	0	0	0	0	2
54	75	F	Negative	Negative	0	0	0	0	0	0	0	0	0	0	1	1	1	0	0	0	0	3
55	75	F	Negative	Negative	0	1	1	1	0	0	0	1	4	0	1	1	1	0	0	0	0	3
56	75	F	Negative	Negative	0	1	0	0	0	0	0	0	1	0	1	0	0	0	0	0	0	1
57	75	M	Negative	Negative	1	1	0	1	0	0	1	0	4	1	1	0	1	0	0	1	0	4
58	76	M	Negative	Negative	1	0	1	1	0	0	0	0	3	1	0	1	1	0	0	0	0	3
59	76	M	Negative	Negative	0	0	0	0	0	0	1	0	1	0	0	0	0	0	0	1	0	1
60	77	M	Negative	Negative	0	0	1	1	1	0	0	0	3	0	0	0	1	1	0	2	0	4
61	77	M	Negative	Negative	0	1	0	0	2	0	2	0	5	1	1	0	0	2	0	2	2	8
62	78	F	Negative	Negative	1	0	1	1	0	0	1	0	4	0	0	0	1	0	0	0	0	1
63	78	M	Negative	Negative	0	0	0	0	0	0	0	0	0	0	0	0	0	0	0	1	0	1
64	78	M	Positive	Negative	1	1	1	1	0	0	1	0	5	1	1	1	1	0	0	1	0	5
65	80	F	Positive	Negative	1	0	0	1	0	0	0	0	2	1	0	1	1	0	0	0	0	3
66	80	M	Positive	Negative	0	1	1	1	1	0	1	0	5	0	1	1	1	1	0	1	0	5
67	81	M	Negative	Negative	0	0	0	1	0	0	0	0	1	1	1	1	2	0	0	2	0	7
68	81	F	Positive	Negative	0	1	0	0	0	0	2	0	3	0	1	1	1	0	0	1	0	4
69	81	M	Negative	Negative	1	1	1	2	0	0	1	0	6	1	1	1	1	0	0	1	0	5
70	81	F	Negative	Negative	1	0	1	1	0	0	0	0	3	1	1	1	1	1	0	1	0	6
71	81	M	Negative	Negative	0	0	0	0	1	0	0	0	1	0	0	0	0	1	0	0	0	1
72	81	F	Positive	Negative	0	0	0	0	0	0	1	0	1	0	0	0	0	0	0	1	0	1
73	82	F	Negative	Negative	0	0	0	0	0	0	0	0	0	0	0	0	0	0	0	0	0	0
74	82	F	Negative	Negative	1	1	1	1	0	1	1	0	6	1	1	1	2	0	0	2	0	7
75	82	F	Negative	Negative	0	1	0	0	0	0	0	0	1	0	1	0	0	0	0	1	0	2
76	83	M	Negative	Negative	0	0	0	0	0	0	0	0	0	0	1	0	1	0	0	1	0	3
77	84	M	Negative	Negative	0	1	1	1	0	0	1	0	4	0	1	1	2	0	0	1	0	5
78	85	F	Negative	Negative	0	0	0	0	0	0	1	0	1	0	0	0	0	0	0	1	0	1
79	85	M	Negative	Negative	0	1	1	1	0	0	0	0	3	0	1	1	2	0	0	0	0	4
80	88	F	Negative	Positive	0	0	1	1	0	0	1	0	3	0	0	0	0	0	1	1	0	2
81	89	M	Negative	Positive	1	1	0	0	0	0	0	0	2	1	1	0	0	0	0	0	0	2
82	90	F	Negative	Negative	1	0	1	0	0	0	2	0	4	1	0	1	1	0	0	2	0	5
83	91	F	Negative	Negative	0	1	2	1	0	0	2	0	6	0	1	2	1	0	0	2	0	6
84	91	F	Positive	Negative	0	0	1	0	0	0	0	0	1	0	0	1	0	0	0	0	0	1
85	100	F	Negative	Negative	1	0	0	0	0	0	0	1	2	2	1	1	1	0	0	2	0	7

GroupNX													
No.	(1) Age	(2)Sex	(3) Oral candidiasis		(4)OHAT (Before)								
			Before	After	Lips	Tongue	Gums	Saliva	Natural teeth	Dentures	Oral cleanliness	Dental pain	Total score
1	42	M	Negative		0	0	0	0	0	0	1	0	1
2	45	M	Negative		2	0	1	1	0	0	0	0	4
3	46	F	Negative		1	0	0	1	0	0	1	0	3
4	49	F	Negative		2	1	1	1	0	0	0	0	5
5	52	M	Negative		1	0	0	1	0	0	1	0	3
6	52	F	Negative		2	0	1	0	0	0	1	0	4
7	61	M	Negative		0	1	1	2	1	0	1	0	6
8	61	M	Negative		0	1	1	1	0	0	0	0	3
9	65	M	Negative		0	0	0	0	0	0	0	0	0
10	68	M	Positive		0	1	1	1	0	1	0	0	4
11	71	F	Positive		1	1	0	0	0	0	1	1	4
12	72	M	Negative		1	0	1	1	0	0	1	0	4
13	73	M	Negative		0	1	1	2	1	0	2	0	7
14	74	M	Negative		1	1	1	1	0	0	1	0	5
15	74	M	Negative		0	1	1	1	0	0	1	0	4
16	74	F	Negative		0	0	0	0	0	0	0	0	0
17	76	M	Negative		1	1	0	0	0	0	0	0	2
18	77	F	Negative		0	0	0	0	0	0	2	0	2
19	77	F	Negative		0	0	0	0	0	0	0	0	0
20	80	M	Negative		0	1	0	0	0	0	1	0	2
21	81	M	Negative		0	0	0	1	0	0	0	0	1
22	81	M	Negative		0	0	0	1	0	2	1	0	4
23	82	M	Negative		2	1	0	1	0	0	1	0	5
24	82	M	Negative		1	1	2	1	0	0	0	0	5
25	83	F	Negative		0	1	0	1	0	0	1	0	3
26	86	M	Negative		0	1	1	1	0	0	0	0	3
27	87	M	Negative		1	1	0	1	0	0	2	0	5

28	88	F	Negative	0	0	0	0	0	0	2	0	2
29	90	M	Negative	0	0	0	0	0	0	1	0	1
30	92	F	Negative	0	1	0	1	0	0	1	0	3
