# Supplementary material for: Longitudinal changes in oral conditions and oral candidiasis in palliative care inpatients: a longitudinal observational study
Source: Front Dent Med. 2026 Jul 2;7:1831411. doi: 10.3389/fdmed.2026.1831411 (PMC13372980; doi:10.3389/fdmed.2026.1831411)
Supplement: Supplementary file 5 [file Datasheet4.pdf]

Table S4 : Details of the oral health of the 138 discharged participants (Group AD).

No.	(1)Age	(2)Sex	(3) Schedule			(4)ADL	(5)Number of remaining teeth (times)	(6)Nutrition route (oral intake)	Oral candidiasis		OHAT (Before)									OHAT (After)								
			a Days	b Times	c Frequency (%)				Before	After	Lips	Tongue	Gums	Saliva	Natural teeth	Dentures	Oral cleanliness	Dental pain	Total score	Lips	Tongue	Gums	Saliva	Natural teeth	Dentures	Oral cleanliness	Dental pain	Total score
1	44	F	9	3	33.3	Independence	28	Possible	Negative	Negative	0	0	0	0	0	0	0	0	0	0	0	0	0	0	0	0	0	0
2	47	F	16	4	25.0	Needs assistance	30	Possible	Negative	Negative	1	1	1	1	1	0	1	0	6	0	1	1	1	1	0	1	0	5
3	47	F	21	4	19.0	Needs assistance	27	Possible	Negative	Negative	1	1	1	1	0	0	0	0	4	0	0	0	0	0	0	0	0	0
4	49	F	24	7	29.1	Independence	27	Possible	Negative	Negative	0	0	0	0	0	0	0	0	0	1	1	0	1	0	0	0	0	3
5	49	F	8	2	25.0	Independence	18	Possible	Negative	Negative	0	0	0	0	0	0	0	0	0	0	0	0	0	0	0	0	0	0
6	49	F	42	7	16.6	Independence	28	Possible	Negative	Negative	1	1	1	1	0	0	1	0	5	1	1	1	1	0	0	0	0	4
7	49	M	55	6	10.9	Needs assistance	29	Possible	Negative	Negative	0	0	0	0	0	0	0	0	0	0	1	0	0	0	0	0	0	1
8	50	M	8	2	25.0	Independence	27	Possible	Negative	Negative	0	0	0	0	0	0	0	0	0	0	0	0	0	0	0	0	0	0
9	50	F	19	3	15.7	Independence	28	Possible	Negative	Negative	0	1	0	0	0	0	1	0	2	0	0	0	0	0	0	1	0	1
10	51	F	10	2	20.0	Independence	29	Possible	Negative	Negative	1	0	0	0	0	0	0	0	1	1	0	0	0	0	0	0	0	1
11	54	M	60	8	13.3	Independence	20	Possible	Negative	Negative	0	0	0	0	0	0	1	0	1	0	0	0	0	2	0	1	0	3
12	55	M	21	4	19.0	Needs assistance	27	Possible	Negative	Negative	0	0	0	0	1	0	0	0	1	0	0	0	0	1	0	0	0	1
13	56	M	27	3	11.1	Independence	26	Possible	Negative	Negative	0	0	0	0	0	0	0	0	0	0	1	0	0	0	0	1	0	2
14	56	F	57	9	15.7	Independence	28	Possible	Negative	Negative	0	0	0	0	0	0	0	0	0	0	0	0	0	0	0	0	0	0
15	56	F	9	3	33.3	Independence	27	Possible	Negative	Negative	0	1	0	0	0	0	1	0	2	0	1	0	0	0	0	0	0	1
16	56	M	3	2	66.6	Needs assistance	27	Possible	Negative	Negative	0	0	0	0	1	0	0	0	1	0	0	0	0	0	0	0	0	0
17	57	M	5	3	60.0	Needs assistance	25	Possible	Positive	Negative	0	1	0	1	0	0	1	0	3	0	1	0	1	0	0	1	0	3
18	58	M	3	2	66.6	Needs assistance	30	Possible	Negative	Negative	0	1	1	1	0	0	1	0	4	0	1	0	0	0	0	0	0	1
19	58	M	8	3	37.5	Needs assistance	28	Possible	Negative	Negative	0	2	0	0	0	0	1	0	3	0	0	0	0	0	0	0	0	0
20	59	M	29	6	20.6	Independence	21	Possible	Negative	Negative	1	1	1	1	0	0	1	0	5	1	1	1	1	0	0	1	0	5
21	59	M	45	3	6.6	Independence	25	Possible	Negative	Negative	0	0	0	0	0	0	1	0	1	0	0	0	0	0	0	0	0	0
22	59	F	19	6	31.5	Needs assistance	29	Possible	Negative	Negative	0	0	0	1	0	0	0	0	1	0	1	0	1	0	0	0	0	2
23	60	M	10	3	30.0	Independence	28	Possible	Negative	Negative	0	1	0	0	0	0	1	0	2	0	0	1	1	0	0	1	0	3
24	60	F	34	4	11.7	Needs assistance	28	Impossible	Negative	Negative	0	1	0	0	0	0	0	0	1	0	0	0	0	0	0	0	0	0
25	62	M	26	8	30.7	Needs assistance	28	Possible	Negative	Negative	0	0	0		0	0	0	0	0	0	1	1	1	0	0	0	0	3
26	62	F	30	5	16.6	Needs assistance	26	Possible	Negative	Negative	0	1	0	0	0	0	0	0	1	0	1	0	0	0	0	0	0	1
27	63	M	59	3	5.0	Needs assistance	25	Possible	Negative	Negative	0	0	0	0	0	0	0	0	0	0	0	0	0	0	0	0	0	0
28	63	M	19	5	26.3	Needs assistance	13	Possible	Negative	Negative	0	1	0	0	0	0	2	0	3	0	0	0	0	0	0	1	0	1
29	64	M	28	7	25.0	Independence	0	Possible	Negative	Negative	0	1	2	1	1	2	2	0	9	0	1	2	1	1	2	2	0	9
30	64	M	59	9	15.2	Independence	26	Possible	Negative	Negative	0	0	0	1	0	0	0	0	1	0	0	0	0	0	0	0	0	0
31	64	M	53	5	9.4	Needs assistance	27	Possible	Negative	Negative	0	0	0	0	1	0	0	0	1	0	0	0	0	1	0	0	0	1
32	64	M	41	6	14.6	Needs assistance	24	Possible	Negative	Negative	0	1	0	1	0	0	1	0	3	0	1	0	0	0	0	0	0	1
33	65	M	7	3	42.8	Needs assistance	27	Impossible	Negative	Negative	1	0	0	0	0	0	0	0	1	1	0	1	1	0	0	1	0	4
34	65	F	9	4	44.4	Needs assistance	22	Possible	Negative	Positive	0	1	1	1	0	0	0	0	3	0	1	1	1	0	0	0	0	3
35	66	M	2	2	100.0	Independence	26	Possible	Negative	Negative	0	1	0	0	0	0	1	0	2	0	1	1	1	0	0	1	0	4
36	66	F	8	2	25.0	Independence	16	Possible	Negative	Positive	0	0	0	0	0	0	0	0	0	0	0	0	0	0	0	0	0	0
37	66	F	6	2	33.3	Independence	28	Possible	Negative	Negative	0	0	0	0	1	0	0	0	1	0	0	0	0	1	0	0	0	1
38	66	M	4	3	75.0	Independence	26	Possible	Negative	Negative	0	1	0	1	0	0	1	0	3	0	1	0	0	0	0	0	0	1
39	66	M	57	10	17.5	Needs assistance	27	Possible	Negative	Negative	0	2	2	0	0	0	1	0	5	0	0	0	0	0	0	0	0	0
40	66	M	11	7	63.6	Needs assistance	27	Possible	Negative	Negative	1	1	1	2	0	0	1	0	6	1	1	1	2	0	0	1	0	6
41	67	F	7	2	28.5	Independence	18	Possible	Negative	Negative	0	0	0	0	0	0	0	0	0	0	0	0	0	0	0	0	0	0
42	67	F	6	2	33.3	Independence	22	Possible	Negative	Negative	1	0	0	0	0	0	0	0	1	0	0	0	0	0	0	0	0	0
43	67	M	36	8	22.2	Independence	23	Possible	Negative	Negative	1	0	2	1	2	0	2	2	10	1	0	2	1	2	0	1	0	7
44	67	F	46	6	13.0	Needs assistance	27	Possible	Negative	Negative	0	0	0	0	0	0	0	0	0	1	1	1	1	0	0	0	0	4
45	67	F	59	4	6.7	Needs assistance	27	Possible	Negative	Positive	0	0	0	1	0	0	2	0	3	0	0	0	1	0	0	0	0	1
46	67	M	57	7	12.2	Needs assistance	8	Possible	Positive	Negative	0	1	1	1	2	0	2	0	7	0	1	0	0	2	0	1	0	4
47	67	M	2	2	100.0	Needs assistance	2	Impossible	Negative	Negative	0	1	1	1	1	0	1	0	5	0	1	1	1	1	0	1	0	5
48	68	M	9	3	33.3	Independence	22	Possible	Negative	Negative	0	1	0	0	0	0	2	0	3	0	1	0	0	0	0	1	0	2
49	68	M	7	3	42.8	Needs assistance	27	Possible	Negative	Negative	0	1	0	0	1	0	1	0	3	0	1	0	0	1	0	1	0	3
50	68	M	16	3	18.7	Needs assistance	27	Possible	Negative	Negative	1	0	0	2	0	0	0	2	5	0	1	0	0	0	0	1	0	2
51	68	F	59	6	10.1	Needs assistance	23	Impossible	Negative	Negative	0	0	0	0	0	0	0	0	0	0	0	0	0	0	0	0	0	0
52	68	M	13	6	46.1	Needs assistance	27	Possible	Positive	Negative	0	2	2	1	0	0	2	1	8	0	0	1	1	0	0	1	0	3
53	69	F	8	2	25.0	Independence	12	Possible	Negative	Negative	0	0	0	0	0	2	0	0	2	0	0	0	0	0	0	2	0	2
54	69	F	31	8	25.8	Needs assistance	22	Possible	Negative	Negative	1	0	0	0	1	0	2	0	4	1	0	0	0	1	0	1	0	3
55	69	F	4	3	75.0	Needs assistance	4	Impossible	Negative	Negative	2	1	2	0	0	0	1	1	7	1	1	1	1	0	0	1	0	5
56	70	M	8	2	25.0	Independence	16	Possible	Negative	Negative	0	1	0	0	2	0	1	0	4	0	1	0	0	2	1	1	0	5
57	70	M	23	6	26.0	Independence	6	Possible	Negative	Negative	1	0	0	0	0	0	1	0	2	0	0	1	1	0	0	1	0	3
58	70	M	15	3	20.0	Independence	19	Possible	Negative	Negative	0	0	0	0	0	0	0	0	0	0	0	0	0	0	0	0	0	0
59	70	M	21	5	23.8	Independence	28	Possible	Negative	Negative	0	1	0	0	0	0	0	0	1	0	1	0	0	0	0	0	0	1
60	70	M	22	3	13.6	Independence	24	Possible	Positive	Negative	0	0	0	0	0	0	1	0	1	0	0	0	0	0	0	0	0	0
61	70	F	18	2	11.1	Independence	26	Possible	Negative	Negative	0	1	0	0	0	0	1	0	2	0	0	0	0	0	0	1	0	1
62	70	F	10	5	50.0	Needs assistance	24	Possible	Negative	Negative	0	0	0	0	0	0	1	0	1	0	0	0	0	1	0	1	0	2
63	70	F	8	3	37.5	Needs assistance	26	Possible	Negative	Negative	1	1	0	2	0	0	1	0	5	0	0	1	0	0	1	0	0	2
64	71	M	29	6	20.6	Independence	26	Possible	Positive	Positive	1	1	1	1	0	0	2	0	6	0	0	1	1	0	0	1	0	3
65	71	F	18	5	27.7	Independence	4	Possible	Negative	Negative	0	0	2	0	0	0	1	0	3	0	0	0	0	0	0	0	0	0
66	71	M	8	3	37.5	Needs assistance	27	Possible	Negative	Negative	0	0	0	0	0	2	0	0	2	0	0	0	0	0	2	0	0	2
67	72	M	16	5	31.2	Independence	19	Possible	Negative	Negative	0	0	0	0	0	0	0	0	0	1	1	1	1	0	0	1	0	5
68	72	M	2	2	100.0	Needs assistance	4	Possible	Negative	Negative	0	1	0	0	0	0	1	0	2	0	1	0	0	0	0	1	0	2
69	72	M	7	2	28.5	Needs assistance	19	Possible	Negative	Positive	1	1	1	1	0	0	1	0	5	0	1	1	1	0	0	2	0	5

70	72	F	3	2	66.6	Needs assistance	14	Possible	Negative	Negative	0	1	1	1	0	0	1	0	4	0	1	1	1	0	0	1	0	4
71	72	M	28	5	17.8	Needs assistance	18	Impossible	Negative	Negative	0	1	0	0	0	1	1	0	3	0	1	0	0	0	0	0	0	1
72	72	F	11	4	36.3	Needs assistance	7	Possible	Negative	Positive	1	0	1	1	0	0	0	1	4	1	0	1	0	0	1	0	0	3
73	73	M	7	4	57.1	Independence	3	Possible	Negative	Negative	0	0	0	0	0	0	0	0	0	0	1	1	1	0	0	1	0	4
74	73	M	8	2	25.0	Independence	6	Possible	Negative	Negative	0	0	0	0	1	0	1	0	2	0	0	0	0	1	0	1	0	2
75	73	M	7	2	28.5	Independence	28	Possible	Positive	Positive	0	0	0	0	0	0	0	0	0	0	0	0	0	0	0	0	0	0
76	73	M	6	3	50.0	Independence	22	Possible	Negative	Negative	0	0	1	1	0	0	0	0	2	0	0	1	1	0	0	0	0	2
77	73	F	9	4	44.4	Independence	2	Possible	Positive	Negative	0	0	0	0	0	0	0	0	0	0	0	0	0	0	0	0	0	0
78	73	F	8	3	37.5	Independence	21	Possible	Negative	Negative	0	0	0	0	2	0	1	0	3	0	0	0	0	2	0	1	0	3
79	73	F	18	2	11.1	Independence	18	Possible	Negative	Negative	0	0	0	0	0	0	1	0	1	0	0	0	0	0	0	0	0	0
80	73	F	12	3	25.0	Needs assistance	28	Possible	Negative	Negative	0	0	0	0	0	0	0	0	0	0	0	0	0	0	0	0	0	0
81	74	F	3	2	66.6	Independence	27	Possible	Negative	Negative	0	0	0	0	0	0	0	0	0	0	0	0	0	0	0	0	0	0
82	74	F	11	5	45.4	Independence	0	Possible	Positive	Negative	0	0	0	0	0	0	0	0	0	0	0	0	0	0	0	0	0	0
83	74	F	13	3	23.0	Independence	9	Possible	Positive	Negative	1	0	0	0	0	0	0	0	1	1	0	0	0	0	0	0	0	1
84	74	F	2	2	100.0	Independence	7	Possible	Negative	Negative	1	1	1	1	0	1	1	0	6	0	1	1	2	0	1	1	0	6
85	75	M	7	2	28.5	Independence	27	Possible	Negative	Negative	0	1	0	0	0	0	1	0	2	0	1	0	0	0	0	1	0	2
86	75	F	13	4	30.7	Needs assistance	18	Possible	Negative	Negative	0	0	0	0	0	0	0	0	0	0	0	0	0	0	0	0	0	0
87	75	M	4	4	100.0	Needs assistance	28	Possible	Positive	Negative	0	0	0	0	0	0	0	0	0	0	1	1	1	0	0	1	0	4
88	75	M	26	6	23.0	Needs assistance	20	Possible	Negative	Negative	0	1	0	0	0	0	1	0	2	0	0	0	0	0	0	0	0	0
89	76	M	20	7	35.0	Independence	18	Possible	Negative	Negative	0	1	0	1	0	0	0	0	2	0	0	0	0	0	0	0	0	0
90	76	F	12	3	25.0	Needs assistance	12	Possible	Negative	Negative	0	1	0	0	0	0	0	0	1	0	0	0	0	0	0	0	0	0
91	76	F	14	4	28.5	Needs assistance	0	Possible	Negative	Negative	0	1	0	0	1	0	1	0	3	0	0	0	0	1	0	1	0	2
92	76	F	15	4	26.6	Needs assistance	11	Impossible	Negative	Negative	0	1	0	0	2	1	2	0	6	0	0	0	0	0	0	0	0	0
93	77	M	6	2	33.3	Independence	28	Possible	Negative	Negative	0	1	1	1	0	0	1	0	4	0	1	1	1	0	0	1	0	4
94	77	M	4	2	50.0	Independence	0	Possible	Positive	Negative	0	0	0	0	0	2	0	0	2	0	0	0	0	0	2	0	0	2
95	77	M	14	14	100.0	Independence	20	Possible	Negative	Negative	0	0	0	0	0	0	1	0	1	0	0	0	0	0	0	0	0	0
96	77	M	14	4	28.5	Needs assistance	10	Possible	Negative	Negative	0	0	1	0	0	0	2	0	3	1	1	1	0	0	0	1	0	4
97	77	M	29	5	17.2	Needs assistance	19	Possible	Negative	Negative	0	0	0	0	0	0	0	0	0	0	0	0	0	0	0	0	0	0
98	77	M	14	4	28.5	Needs assistance	16	Possible	Negative	Negative	0	1	1	1	0	0	1	0	4	0	1	1	1	0	0	1	0	4
99	77	M	22	4	18.1	Needs assistance	28	Impossible	Negative	Negative	0	1	0	0	0	0	1	0	2	0	1	0	0	0	0	1	0	2
100	78	M	3	2	66.6	Needs assistance	6	Possible	Negative	Negative	0	0	1	1	2	2	1	0	7	0	0	1	1	2	2	0	0	6
101	78	F	2	2	100.0	Needs assistance	28	Possible	Negative	Negative	2	0	0	0	0	0	0	0	2	0	0	0	0	0	0	0	0	0
102	78	M	5	2	40.0	Needs assistance	24	Possible	Negative	Positive	0	0	0	0	0	0	1	0	1	0	0	0	0	0	0	1	0	1
103	79	M	37	4	10.8	Independence	0	Possible	Negative	Negative	0	0	0	0	0	0	0	0	0	0	0	0	0	0	0	0	0	0
104	79	M	20	3	15.0	Needs assistance	17	Possible	Negative	Positive	0	0	0	0	0	0	1	0	1	0	1	1	1	0	0	1	0	4
105	79	F	26	4	15.3	Needs assistance	7	Possible	Negative	Negative	0	0	0	0	0	0	0	0	0	0	0	0	0	0	0	0	0	0
106	80	F	13	4	30.7	Independence	26	Possible	Negative	Negative	0	1	0	0	0	0	0	0	1	0	0	0	0	0	0	1	0	1
107	80	M	10	2	20.0	Needs assistance	5	Possible	Negative	Negative	0	0	1	1	0	0	2	0	4	0	0	1	1	0	0	2	0	4
108	81	M	20	4	20.0	Independence	22	Possible	Negative	Negative	0	0	0	0	0	0	0	0	0	0	1	0	0	0	0	0	0	1
109	81	F	4	2	50.0	Needs assistance	28	Possible	Negative	Negative	0	0	0	0	0	0	0	0	0	0	1	1	1	0	0	1	0	4
110	81	F	12	5	41.6	Needs assistance	6	Possible	Negative	Negative	0	0	0	1	0	0	0	1	2	0	1	1	1	0	2	1	0	6
111	81	M	10	2	20.0	Needs assistance	17	Possible	Negative	Negative	2	1	1	2	0	0	2	0	8	2	1	1	2	0	0	2	0	8
112	82	F	5	2	40.0	Independence	12	Possible	Negative	Negative	0	1	0	0	0	0	0	0	1	0	1	0	0	0	0	0	0	1
113	82	M	31	8	25.8	Independence	27	Possible	Negative	Negative	0	1	0	0	0	0	2	0	3	0	0	0	0	0	0	2	0	2
114	82	F	7	4	57.1	Needs assistance	2	Impossible	Negative	Negative	1	1	1	2	0	0	1	0	6	1	1	2	2	0	0	2	0	8
115	82	F	59	6	10.1	Needs assistance	4	Possible	Negative	Negative	0	1	0	0	0	0	0	0	1	0	1	0	0	0	0	0	0	1
116	82	F	8	2	25.0	Needs assistance	4	Possible	Negative	Negative	0	0	0	0	0	0	0	0	0	0	0	0	0	0	0	0	0	0
117	83	M	8	2	25.0	Independence	22	Possible	Negative	Negative	0	1	0	0	0	0	0	0	1	0	1	0	0	0	0	0	0	1
118	84	M	11	3	27.2	Independence	27	Possible	Negative	Negative	0	0	0	0	0	0	1	0	1	0	0	0	0	0	0	1	0	1
119	84	M	27	8	29.6	Independence	27	Possible	Negative	Negative	0	0	0	0	0	0	1	1	2	0	0	0	0	0	0	1	0	1
120	84	M	36	8	22.2	Needs assistance	26	Possible	Negative	Negative	0	0	0	0	0	0	0	0	0	0	0	0	0	0	0	0	0	0
121	85	F	14	4	28.5	Independence	7	Possible	Negative	Positive	1	0	0	0	0	0	0	0	1	1	2	0	1	0	0	1	0	5
122	85	M	14	3	21.4	Independence	26	Possible	Negative	Negative	0	0	0	0	0	0	0	0	0	0	0	0	0	0	0	0	0	0
123	85	F	7	3	42.8	Independence	0	Possible	Negative	Negative	0	0	0	0	0	0	0	0	0	0	0	0	0	0	0	0	0	0
124	85	M	13	4	30.7	Independence	27	Possible	Positive	Negative	0	1	0	0	0	0	0	0	1	0	0	0	0	0	0	0	0	0
125	85	F	5	3	60.0	Needs assistance	18	Possible	Negative	Negative	1	1	1	1	1	0	2	0	7	0	0	1	1	1	1	1	0	5
126	85	F	8	3	37.5	Needs assistance	4	Possible	Positive	Negative	1	1	0	1	1	0	1	0	5	0	0	0	0	0	0	0	0	0
127	86	M	4	2	50.0	Needs assistance	0	Possible	Negative	Positive	0	0	0	0	1	0	1	0	2	0	0	0	0	1	0	1	0	2
128	86	F	7	3	42.8	Needs assistance	8	Possible	Negative	Negative	0	0	0	0	0	2	0	0	2	0	0	0	0	0	0	0	0	0
129	86	M	14	2	14.2	Needs assistance	21	Impossible	Negative	Negative	0	0	0	0	0	0	0	0	0	0	0	0	0	0	0	0	0	0
130	87	F	15	3	20.0	Independence	9	Possible	Negative	Negative	0	0	0	0	0	0	1	0	1	0	0	0	0	0	0	1	0	1
131	89	M	2	2	100.0	Needs assistance	17	Possible	Negative	Negative	1	0	0	0	0	0	0	0	1	0	0	1	0	1	0	1	1	4
132	89	M	28	4	14.2	Needs assistance	28	Possible	Positive	Negative	0	1	1	1	0	0	1	0	4	0	1	1	1	0	0	1	0	4
133	89	M	3	2	66.6	Needs assistance	15	Possible	Positive	Positive	0	1	1	0	0	0	2	0	4	0	0	0	0	0	0	0	0	0
134	89	F	28	5	17.8	Needs assistance	1	Possible	Positive	Negative	0	2	1	2	0	0	1	0	6	0	0	0	0	0	0	0	0	0
135	89	M	14	6	42.8	Needs assistance	25	Possible	Negative	Negative	0	1	1	1	0	0	1	0	4	0	1	1	1	0	0	1	0	4
136	91	M	6	2	33.3	Needs assistance	4	Possible	Negative	Negative	0	1	0	0	0	0	1	0	2	0	1	0	0	0	0	1	0	2
137	91	F	2	2	100.0	Needs assistance	4	Possible	Negative	Negative	0	1	0	0	1	0	1	0	3	0	1	0	0	1	0	1	0	3
138	95	M	7	3	42.8	Independence	28	Possible	Negative	Negative	0	0	0	0	0	0	0	0	0	0	0	0	0	0	0	0	0	0
